# Mathematical modelling of lymphatic filariasis elimination programmes in India: required duration of mass drug administration and post-treatment level of infection indicators

**DOI:** 10.1186/s13071-016-1768-y

**Published:** 2016-09-13

**Authors:** Purushothaman Jambulingam, Swaminathan Subramanian, S. J. de Vlas, Chellasamy Vinubala, W. A. Stolk

**Affiliations:** 1 Vector Control Research Centre (Indian Council of Medical Research), Indira Nagar, Puducherry, 605006 India; 2 Department of Public Health, Erasmus MC, University Medical Centre Rotterdam, Rotterdam, The Netherlands

**Keywords:** Lymphatic filariasis, *Wuchereria bancrofti*, *Culex quinquefasciatus*, India, Individual-based model, Mass treatment, Diethylcarbamazine and albendazole, Elimination, Prevalence, MDA duration, Post-MDA residual infection

## Abstract

**Background:**

India has made great progress towards the elimination of lymphatic filariasis. By 2015, most endemic districts had completed at least five annual rounds of mass drug administration (MDA). The next challenge is to determine when MDA can be stopped. We performed a simulation study with the individual-based model LYMFASIM to help clarify this.

**Methods:**

We used a model-variant for Indian settings. We considered different hypotheses on detectability of antigenaemia (Ag) in relation to underlying adult worm burden, choosing the most likely hypothesis by comparing the model predicted association between community-level microfilaraemia (Mf) and antigenaemia (Ag) prevalence levels to observed data (collated from literature). Next, we estimated how long MDA must be continued in order to achieve elimination in different transmission settings and what Mf and Ag prevalence may still remain 1 year after the last required MDA round. The robustness of key-outcomes was assessed in a sensitivity analysis.

**Results:**

Our model matched observed data qualitatively well when we assumed an Ag detection rate of 50 % for single worm infections, which increases with the number of adult worms (modelled by relating detection to the presence of female worms). The required duration of annual MDA increased with higher baseline endemicity and lower coverage (varying between 2 and 12 rounds), while the remaining residual infection 1 year after the last required treatment declined with transmission intensity. For low and high transmission settings, the median residual infection levels were 1.0 % and 0.4 % (Mf prevalence in the 5+ population), and 3.5 % and 2.0 % (Ag prevalence in 6–7 year-old children).

**Conclusion:**

To achieve elimination in high transmission settings, MDA must be continued longer and infection levels must be reduced to lower levels than in low-endemic communities. Although our simulations were for Indian settings, qualitatively similar patterns are also expected in other areas. This should be taken into account in decision algorithms to define whether MDA can be interrupted. Transmission assessment surveys should ideally be targeted to communities with the highest pre-control transmission levels, to minimize the risk of programme failure.

**Electronic supplementary material:**

The online version of this article (doi:10.1186/s13071-016-1768-y) contains supplementary material, which is available to authorized users.

## Background

The fact that humans are the only reservoir host for lymphatic filariasis (LF), together with the availability of simple, safe, and inexpensive drugs for treatment and effective diagnostic tools, led to the recognition that LF might be eradicable [1]. The Global Programme to Eliminate Lymphatic Filariasis (GPELF) was launched in 2000, aiming to eliminate LF as a public health problem by 2020 [2]. The recommended strategy is to treat entire at-risk populations annually through mass drug administration (MDA) with a single dose of ivermectin and albendazole (IVM + ALB) in sub-Saharan Africa or with diethylcarbamazine and albendazole (DEC + ALB) in other regions, including India, for a minimum of 5 years with effective population coverage of treatment [1, 3].

India has made great progress towards the elimination of lymphatic filariasis. By 2015, most endemic districts have completed the WHO recommended minimum of five annual effective (i.e. at least 65 % treatment coverage) rounds of MDA with the diethylcarbamazine-albendazole drug-combination (DEC + ALB) [4]. The key challenge now is to determine whether this effort has been sufficient to interrupt transmission, so that MDA can safely be stopped in all treated areas [5, 6]. In *W. bancrofti* endemic areas where the main vector is *Anopheles* or *Culex*, the critical threshold below which MDA can be stopped is assumed to be 1 % for microfilaraemia (Mf) prevalence and 2 % for antigenaemia (Ag) prevalence [3]. Lower values (0.5 % and 1 %) are proposed for *Aedes*-transmitted infection. Five rounds of annual MDA may not always be sufficient to break transmission, e.g. if coverage is compromised or in settings with the highest transmission intensity. Effective monitoring and evaluation are essential to assess whether elimination programmes are on track and whether infection levels have been brought below the critical threshold.

To monitor whether the Mf and Ag prevalence levels are declining as expected, the World Health Organization (WHO) suggests that epidemiological investigations are done in sentinel and spot check sites. A two-step approach has been recommended for deciding when to stop interventions [3, 7]. When a region has completed at least 5 rounds of MDA with sufficient coverage and the Mf or Ag prevalence in sentinel and spot check sites is shown to be below 1 % or 2 % respectively, a standardized “Transmission Assessment Survey” (TAS) should be done to confirm that interventions have reduced the infection levels below a critical threshold [3]. This involves assessing the incidence of infection in 6–7 year-old children. This age-class was chosen because these children already experience exposure to vector bites, but should have been protected from LF infection if MDA were successful in interrupting transmission. The operational feasibility, assumptions and accuracy of TAS have been evaluated in different endemic settings and the general sampling strategy was proven to be feasible and robust [7, 8]. In India, besides assessing Mf or Ag-prevalence in sentinel and spot check sites, Mf-prevalence is determined in 10 randomly selected sites to decide about conducting TAS [9]. In all 10 sites, Mf-prevalence should be below 1 % for the area to conduct a TAS. However, questions remain about the critical thresholds level of infection and the accuracy of TAS to identify areas that move to elimination or still have sufficient LF transmission that will cause resurgence of infection [7].

Mathematical models of lymphatic filariasis transmission and control provide useful tools to identify the conditions under which elimination could be achieved and to estimate critical thresholds of infection [10–12]. In this study, we use the established individual-based model LYMFASIM [13–17]. This model accounts for several factors that are critical for predicting elimination through MDA, including individual heterogeneities in exposure to mosquito biting and compliance with MDA, stochastic effects contributing to elimination/recrudescence, and variability in diagnostic test outcomes in epidemiological surveys. The purpose of our study is to assess the required duration of MDA to achieve elimination and the associated 1-year post-treatment values of Mf and Ag prevalence associated with successful elimination, both for the community as a whole and for 6–7 year children only. This is done for Indian settings, where *Wuchereria bancrofti* is transmitted by *Culex quinquefasciatus*, considering different pre-control endemicity levels.

## Methods

### The LYMFASIM simulation model

#### Model structure

LYMFASIM is an individual-based model for simulating lymphatic filariasis (LF) transmission and control in a dynamic human population [10, 13, 18]. It employs the technique of stochastic micro-simulation [19]. The computer program provides a flexible modelling framework, allowing for the specification of different model variants by adjusting assumptions and parameter values. Below, we provide a brief description of main model characteristics and the simulation of mass drug administration. A complete mathematical description is provided elsewhere [13, 14].

The model simulates a dynamic human population and transmission of infection between human individuals by a mosquito population. The human population consists of a discrete number of individuals and the population composition changes over time due to birth, aging and death of individuals. Humans can be populated by worms (immature or mature, male or female). The lifespan of worms is described by a Weibull distribution with an average duration of 10.2 years, independent of the gender of worms. The duration of the immature period is fixed at 8 months for all worms. During their reproductive period, adult female worms are assumed to produce Mf at a constant rate (“Mf-production rate”, expressed as the produced number of Mf per female worm per month per 20 μl of peripheral blood) if at least one adult male worm is present in the same host. The mosquito biting rate varies between individuals, both as a function of age (increasing linearly with age from a low level to a stable maximum that is reached at the age of 20) and randomly between individuals (with an individual’s exposure assumed to be constant over his lifetime). When a mosquito bites, infection may be transferred from human to mosquito. Exposure heterogeneity causes variation in the number of L3 larvae to which individuals are exposed per month, and hence in the new worm acquisition rate and resulting accumulated worm load. Only a small proportion of the L3 larvae that are released by biting mosquitoes will develop successfully into adult worms (success ratio), and the success ratio may be further reduced if a host has acquired protective “anti-L3” immunity against larvae, as explained further below. The transmission of infection from human to mosquito is governed by a non-linear relationship between Mf intensity in human blood and the average number of infective stage (L3) larvae that will develop in mosquitoes after taking a blood meal. The mean infection level in the mosquito population is derived from the individuals’ Mf density in the blood and exposure to mosquito bites.

#### Parameter values of core biological parameters

For the current analysis, we used the “anti-L3 immunity” LYMFASIM model variant for bancroftian filariasis transmitted by *Culex quinquefasciatus* as previously developed by Subramanian et al. [14], with associated derived parameter values. (The two other model variants developed by Subramanian et al. were not considered: the model without immunity failed to explain the age-patterns of infection, and the anti-fecundity model - which suggests that moderate Mf prevalence levels in human adults may be associated with very high prevalence of adult worm - does not match with our current understanding of the adult worm biology based on antigen prevalence data.) In the anti-L3 immunity model, a person’s level of acquired immunity is dependent on his cumulative exposure to L3 larvae, and immunity protects against establishment of new infections by reducing the probability that new larvae survive to develop into adult worms. The level of protective immunity varies between individuals due to differences in past exposure to infection and random variation between individuals in their ability to develop immunity against L3 larvae.

The core biological parameters of the model with anti-L3 immunity were previously quantified by fitting the model to longitudinal entomological and epidemiological data from an integrated vector management programme carried out in Pondicherry, India, from 1981–1986 [14]. The resulting model fitted well to the data, and also provided accurate estimates of trends in infection prevalence both before and after cessation of integrated vector management. We used the same parameter quantification for the current analysis, simulating settings without integrated vector management programmes. Only the monthly biting rate was varied, to have the model represent sites with different baseline endemicity and transmission conditions. A complete overview of all model assumptions and parameter values as used for this study is provided in Additional file 3.

#### Assumptions about MDA: coverage, compliance and drug efficacy

MDA can be simulated at specified time points. In each round of MDA, a proportion of the total population (defined by the specified coverage) is assumed to be treated with DEC + ALB, the recommended treatment regimen for India. Treatment with DEC + ALB was assumed to kill 70 % of Mf, based on the relative reduction in mean Mf intensity observed 15–30 days after treatment in clinical trials (reviewed elsewhere [20]). In addition, we assumed that treatment kills 65 % of adult worms. A high macrofilaricidal effect is consistent with the sustained reductions in mean Mf intensity that are seen in clinical trials [20, 21], and is backed up by evidence from ultrasonography studies demonstrating a loss of motility following treatment [22, 23], although uncertainty remains on the quantitative efficacy estimates. Death of Mf and adult worms was assumed to occur shortly after treatment (within one month). We further assumed that there is no inter-individual variation in treatment effects and that treatment efficacy is independent of the number of past treatments.

The assumed treatment coverage was varied between scenarios (50 %, 65 % or 80 %), where coverage is defined as the percentage of people taking treatment (i.e. swallowing the drug) out of the total population. The percentage coverage was assumed to be constant over subsequent rounds of MDA. Individual compliance with offered treatment was simulated as a partially systematic process, i.e. it is neither completely random (where each person has the same chance to get treated in each round) nor completely systematic (where all individuals either take all or none of the treatments), but somewhere in between [24]. The simulated proportion of systematic non-compliers (i.e. those who never take treatment) for a given number of treatment rounds is not fixed; it depends on overall treatment coverage levels; the proportion of systematic non-compliers in the total population increases when the overall coverage declines, and vice versa. This partially systematic process represented the compliance pattern of a MDA programme for LF in Tamil Nadu, India [25] and onchocerciasis in Asubende, Ghana, very well [24], and we assume that compliance patterns for LF treatment are similar. Variation between age and sex groups in compliance was not considered.

#### Simulation output

The model keeps track of changes in infection status (e.g. number of immature and mature, male and female worms) at the individual level over time. Simulation output contains the results of simulated epidemiological surveys, to be performed at user-defined moments (calendar year and month) and three types of output can be requested: 1) summary output at population level; 2) detailed output at population level by age and sex; 3) individual-level output. The latter provides information on the number of male and female worms per individual, and through further analysis of these output data the user can derive population-level indicators. For this study, we were mainly interested on output on Mf and Ag prevalence by age and sex. We simulated a population consisting of 3750 people on average at the moment of first MDA; the population gradually grows over time with a rate of 1.9 % per year. The model allows for measurement variation in simulated Mf counts at individual level, thereby also allowing for false-negative Mf counts. The presence of antigenaemia is not an explicit part of the model output, but is derived from output on the presence of worms based on a hypothesized association between these two indicators. The first step in this simulation study was to test three alternative hypotheses for this association and determine which fits best to empirical data. This is described further below (step 1 of the simulation study).

### Simulation study design

Our study can be distinguished into four different steps: 1) modelling Ag prevalence; 2) estimating the required duration of mass treatment for achieving elimination; 3) assessing the 1-year post-treatment levels for Mf and Ag prevalence that are associated with successful elimination; and 4) a sensitivity analysis to assess the effect of varying treatment efficacy and timing of assessing residual infection post-MDA.

#### Step 1: Modelling antigenaemia prevalence

The rapid format immunochromatographic card test (ICT) for antigen (Ag) detection [26] is now routinely used in many ongoing elimination programmes for mapping, monitoring progress and deciding when to stop treatment [27]. According to the operational use of Ag testing, we consider an individual’s Ag status as a binary outcome, i.e. individuals are either Ag positive or negative. The detected antigens are thought to originate from adult *Wuchereria bancrofti* parasites [28–30] and antigen tests can demonstrate the presence of adult worm infection in infected people who do not have detectable Mf levels in their blood (e.g. [31–34]). Other modellers assumed that any adult worm would always be detected by the antigen test (as in our hypothesis 1, see below) [11]. However, there is ample evidence that the ICT card test sensitivity is less than 100 %, e.g. from studies demonstrating that antigenaemia can be undetectable in men with ultrasound-detected adult worm nests [35] and from studies showing that the ICT card test detects less infections than other antigen diagnostic tests such as Og4C3 ELISA [36–38] and the Alere Filariasis Test Strip [39, 40]. Some uncertainty still remains regarding the exact source of antigens, the relative contribution of different parasite life stages (male worms, female worms, Mf) to antigenaemia levels, and the test sensitivity for the detection of amicrofilaraemic adult worm infections [41].

In view of prevailing uncertainties, we tested three hypotheses for the association between antigenaemia and the presence of adult worms against empirical data, namely:

- Hypothesis 1: Antigenaemia is assumed to be detectable if at least one male or female worm is present in the host, i.e. we have a perfect diagnostic test with 100 % sensitivity for the detection of all adult worms;
- Hypothesis 2: The antigenaemia detection rate is assumed to increase with the number of adult worms. We simulate this by relating the detectability of antigenaemia to worm sex, assuming that antigenaemia is only detected in the presence of at least one female worm or worm pair; single-sex infections with male worms only remain undetected. This is not implausible: male worms may contribute less to antigenaemia than their female counterparts, as was observed for a related parasite species *Dirofilaria immitis* [41], e.g. due to gender-related processes or simply the larger size of female worms [42]. Since male and female worms in our model occur with the same probability and are independently distributed over human hosts, linking detectability to female worms implies that antigens are detected in 50 %, 75 %, 87.5 %, … of individuals infected with 1, 2, 3, … adult worms, and antigenaemia is always detected in the presence of a male + female worm pair.
- Hypothesis 3: Antigenaemia is detectable only in the presence of at least one male + female worm pair. Since the model assumes that all female worms produce Mf in the presence of a male worm, antigenaemia would mostly concur with microfilaraemia (unless a female worm’s fecundity is reduced by past treatment).

To test the validity of the three hypotheses, we first compared the model-predicted pre-control association between Mf and Ag prevalence to data obtained from literature. We searched the Medline (PubMed) database to identify scientific articles providing pre-control community-level data on both Mf and Ag prevalence. Studies had to present data at community level, but we allowed some variation with respect to the minimum age considered (studies with a minimum age > 10 years were excluded). We did not impose additional selection criteria concerning the diagnostic tools used to measure Mf or Ag prevalence. Although our main interest was in data from the Asian region, we also included data from other regions (to understand the geographic stability of the association, and for future usage by ourselves and others). The literature data are provided in Additional file 4, along with information on search and selection criteria. A scatterplot was created to visualize the association between the two infection indicators in the observed data, for the range of prevalence levels observed in Asian settings (the observed Mf prevalence seldom exceeds 20 %). Model-predicted values of Mf and Ag prevalence for the three hypotheses were overlaid on the scatterplot for visual assessment of the goodness-of-fit of the three hypotheses to the empirical data. To capture some of the between-study variation in the data, model-predictions were made with varying assumptions regarding diagnostic test accuracy (reflecting Mf counts by microscopic examination of either 40 or 60 μl blood, as used in Asian studies) and resulting prevalences are given either for the whole population aged 5 years and above, or are age-standardized to reflect sampling with underrepresentation of children under 10 and of elderly individuals. Simulations were done for an average population size of about 3750 individuals (range 2450–5250 individuals). The goodness of fit of model to data is visually examined.

As a second step, we tested whether the predicted antigen prevalence after several rounds of MDA is also in the right order of magnitude. For this analysis, we used data on Mf and Ag prevalence from a large-scale study that evaluated the impact of 8 annual rounds of MDA in two primary health centres in Thanjavur district in India. Detailed pre-control data were not available from this area, but the district was known to be low-endemic. MDA with DEC alone was given in 1997, 1999, 2000, and 2004; MDA with the combination DEC + ALB was given in 2001, 2002, 2003, and 2007. Coverage achieved was low as indicated by both reported and surveyed coverage [8]. The overall Mf prevalence in the district was still 2.6 % in 2000 as observed in sentinel sites, but was reduced to < 1 % in the 8 sentinel and spot-check sites that have been surveyed since the 2004 round of MDA; data on Ag prevalence were not available from these sites, so we could not overlay observed data on model-predicted trends in Ag-prevalence. We tested the model against the detailed data on Mf and Ag prevalence that were collected in 2008, i.e. one year after the last round of MDA; the data covered a total of 80 villages and 15 urban wards [8]. Model parameters were quantified as described above. We fixed the monthly biting rate at 1600 bites per month per adult male, a value that was known to result in low baseline endemicity in simulated Indian settings. Treatment efficacy parameters for DEC and DEC + ALB were also fixed at previously used values [43]. Next, we tuned the overall treatment coverage (proportion of people treated out of the whole population) to reproduce the observed post-treatment overall Mf prevalence levels in the adult population after 8 rounds of MDA. In view of the low prevalence and wide confidence intervals around age-group specific Mf prevalence, we aggregated the data from the different communities and wards and we did not aim to reproduce age-specific Mf prevalence levels exactly. After confirming that the overall Mf prevalence in adults was adequately reproduced, we tested whether model-predicted patterns of Ag prevalence by age were also in agreement with the data. In view of the many uncertainties involved, we restricted to a qualitative analysis. This was done for each of the three hypotheses about the association between presence of parasites and antigenaemia; see Table 1 for more details regarding the simulated scenarios and used model outputs. The hypothesis that matched best to data in both comparisons was taken as our baseline model for predicting Ag prevalence levels.

**Table 1:** Overview of simulated scenarios and simulation outputs considered, by specific objective

Step	Specific objective	Inputs varied	Output considered
1	Comparison of the model-predicted association between Mf and Ag prevalence at community level to observed data from literature	Hypotheses for modelling Ag-prevalence: 1-3	Model-predicted Mf and Ag prevalence for the population aged 5 years and above, for each run separately
		Mbr: 1500, 1600, 1700, …4000	
	Comparison of the model-predicted post-MDA outcomes to empirical data from Thanjavur district, for the Ag prevalence by age-group	Hypotheses for modelling Ag-prevalence: 1-3	Model-predicted Mf and Ag prevalence by age-group, averaged over multiple repeated runs
		Mbr: 1600	
		Treatment coverage defined by fitting the model to observed patterns of Mf prevalence	
		Efficacy of single treatment with DEC (kills Mf: 70 % and kills adult worm: 50 %) [87]	
		Efficacy of single treatment with DEC + ALB (kills Mf: 70 % and kills adult worm: 65 %) [20]	
2	Assess the required duration of MDA for achieving elimination	Hypotheses for modelling Ag-prevalence: not relevant for this part of the work	Proportion of runs that resulted in elimination (elimination was said to occur if the Mf prevalence was zero, 60 years after the first treatment round)
		Mbr: 1600, 1950, 2200, 2700	
		Treatment duration: varied	
		Treatment coverage: 50 %, 65 %, 80 %	
		Efficacy of single treatment with DEC + ALB (kills Mf: 70 % and kills adult worm: 65 %)	
3	Assess the 1-year post-treatment values for Mf and Ag prevalence associated with successful control, for the community as a whole and for 6–7 year children.	Hypotheses for modelling Ag-prevalence: hypothesis 2 (identified as best in step 1)	Model-predicted Mf and Ag prevalence for the entire population aged 5 years and above and for 6–7 year-old children, as measured 1 year after the last treatment round
		Mbr: 1600, 1950, 2200, 2700	
		Treatment duration: as need to achieve ≥ 99 % probability (estimated in step 2)	
		Treatment coverage: 50 %, 65 %, 80 %	
		Efficacy of single treatment with DEC + ALB (kills Mf: 70 % and kills adult worm: 65 %)	
4	Sensitivity analysis	Hypotheses for modelling Ag-prevalence: hypothesis 2 (identified as best in step 1)	Model-predicted Mf and Ag prevalence for the entire population aged 5 years and above and for 6–7 year-old children, as measured 1 year after the last treatment round, or 6 months or 2 years after treatment
		Mbr: 2200	
		Treatment duration: as need to achieve ≥ 99 % probability (re-estimated)	
		Treatment coverage: 65 %	
		Efficacy of single treatment with DEC + ALB: varied or as in step 3	

#### Step 2: Estimating the required duration of MDA for eliminating LF

We simulated trends in different infection indicators during and after MDA, for four epidemiological settings varying with respect to mean biting rate and baseline endemicity. As baseline prevalence, we took the prevalence that is achieved after a 130-year warming-up period, and just before the first round of MDA. A 130-year warming-up period was necessary to allow the population composition and endemicity levels to stabilize. Simulations were done for the Pondicherry setting, for which the model was originally quantified [14], and three hypothetical settings, which only differed from Pondicherry with respect to the monthly biting rates of mosquitoes and hence the endemicity levels at baseline. The monthly biting rate (mbr) in Pondicherry was 2200, corresponding to a pre-control Mf prevalence of 8.5 % on average. The hypothetical settings reflected communities with low transmission (mbr = 1600, mean baseline Mf prevalence 4.9 %), medium transmission (mbr = 1950, mean baseline Mf prevalence 7.4 %), and high transmission (mbr = 2700, mean baseline Mf prevalence 10.0 %). The indicated biting rates are average biting rates for adults; see Additional file 3 for information regarding associated biting rates in children and variability in exposure between individuals). The predicted Mf prevalence takes account of measurement variation in Mf counts and the possible occurrence of false-negative Mf counts, as would also occur in field situations. We assumed that Mf counts were done by microscopic examination of a 20-μl thick smear of night finger-prick blood, and that the variation in Mf counts in blood smears for an individual follows a negative binomial distribution, similar to Subramanian et al. [14].

Using similar methods as described elsewhere [17] and further described below, we determined the minimum number of MDA rounds that is required to achieve elimination, for each of the four epidemiological settings and for three levels of treatment coverage (50 %, 65 %, 80 %). For each of the 12 epidemiological setting-coverage combinations, we simulated the expected trends in infection during and after MDA, for different durations of MDA (1, 2, 3, … rounds), with 1000 repeated runs per duration to capture the stochastic variations between runs - all with the exact same input assumptions. We recorded for each run whether elimination was eventually reached and for each scenario (combination of epidemiological setting, coverage and duration) we calculated the elimination probability per scenario as the percentage of runs that reached this outcome, with elimination defined as zero Mf prevalence 60 years after the start of MDA (and therefore at least 45 years after the last round of MDA, depending on the simulated number of treatment rounds). For each of the 12 epidemiological setting-coverage combinations, the required duration of MDA was estimated as the lowest number of MDA rounds that resulted in a ≥ 99 % probability of elimination. For this part of the simulation study, we only required the following simulation output per run: the baseline Mf prevalence after a 130-year warming-up period (needed to remove failed runs) and the Mf prevalence 60 years after the first treatment. Other output was not stored. Failed runs (in which the parasite population went to extinction during the warming-up period) were discarded and replaced by news runs, until we had 1000 successful runs in total per scenario. Failure only occurred in the low transmission scenario (mbr = 1600), for about 40 % of the runs.

#### Step 3. Assessing the residual infection prevalence 1-year post-MDA after required treatment duration

Next, for each of the 12 epidemiological setting-coverage combinations, we did a new series of simulation runs with the number of annual MDA rounds specified as required to achieve ≥ 99 % probability of elimination. More output was stored to enable a more detailed assessment of predicted trends in infection and to assess how much residual infection remained 1 year after the last annual treatment round. We did 300 repeated runs per scenario; failed runs were discarded without replacing them with new runs, because the number of remaining successful runs is still sufficient to assess the frequency distribution. The infection indicators of interest were the simulated Mf and Ag prevalence, for the population aged 5 years and above and for 6–7 year-old children, respectively. Ag prevalence was assessed under the hypothesis that matched best to both types of data in step 1 of the work.

#### Step 4. Sensitivity analysis

In a sensitivity analysis, we assessed the influence of modifying assumptions about (1) treatment efficacy and (2) the assumed time interval between the last treatment and the epidemiological assessment. Results are shown for the Pondicherry setting, assuming 65 % coverage of MDA. First, we assessed the influence of treatment efficacy assumptions on the required duration of MDA for achieving elimination and on residual infection levels as measured one year after the last MDA, both for the Mf prevalence at community level (population above 5 years old) and Ag prevalence in 6–7 year-old children. Assumptions were modified as follows:

(i) The fraction of adult worms killed due to treatment was varied at two levels: 50 % and 80 % (*versus* 65 % at baseline), while the fraction of Mf killed was kept at its baseline value (70 %).
(ii) Similarly, the fraction of Mf killed due to treatment was varied at two levels: 40 % and 100 % (*versus* 70 % at baseline), while the fraction of adult worms killed was kept at its baseline value (65 %).

Next, we examined how residual infection levels are influenced by the time interval between the last treatment and the epidemiological assessment. The time interval was halved (6 months) or doubled (2 years). Treatment efficacy parameters were kept at their baseline values (65 % adult worm killed, 70 % Mf killed) and the required duration of MDA was as estimated under step 2.

## Results

### Modelling antigenaemia prevalence

**Fig. 1:**
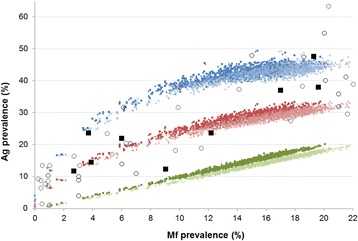
Comparison of the model-predicted association between Mf and Ag prevalence at community level to observed data from literature from Asian settings (black squares) and other regions including Africa, Oceania and the Americas (open black circles). The scale of the horizontal axis is restricted based on the observed values from Asian settings. Coloured dots show the model-predicted Mf and Ag prevalence, which were obtained by varying the average monthly biting rate between 1500–4000 bites per adult person per month. The model predicted Ag prevalence is shown for three different hypotheses on the mechanistic association between the presence of adult worms and detectability of antigenaemia. Hypothesis 1: antigenaemia is detectable in the presence of at least one male or female worm (blue). Hypothesis 2: the Ag detection rate is 50 % for single worm infections, but increases with the number of adult worms, simulated by assuming that antigenaemia is only detectable in the presence of at least one female worm or worm pair (red). Hypothesis 3: antigenaemia is detectable in the presence of at least one male + female worm pair (green). The darker and lighter colours show the association if Mf prevalence is measured in 40 and 60 μl blood, respectively. Simulated prevalence was for the whole population aged 5 years and above (triangles) or was standardized to give the expected prevalence in a study sample in which children under 10 and elderly individuals (squares) are underrepresented. With these provisions, the model captures the entire range of observed Mf prevalence levels in Asian settings

Figure 1 shows the qualitative level of agreement between the model-predictions and collated data from literature on the association between Mf and Ag prevalence at community level, for the range of prevalence levels observed in Asian settings. The number of observations from Asian settings was limited (black squares), but the empirical association is confirmed by observed data from other regions (Africa, Oceania, Americas; open cirles). The model captures the entire range of observed Mf prevalence levels in the Asian region. Model-predicted Ag prevalence levels are generally too high under hypothesis 1 and too low under hypothesis 3. Hypothesis 2 is most compatible with the data, even though the predicted prevalence at higher prevalence levels may be a bit low. The observed data show considerable variation around the model-predicted values, which can be explained by sampling variation due to relatively small sample sizes in the data compounded by variation in the age-composition of the study sample and geographic variation in underlying transmission conditions.

**Fig. 2:**
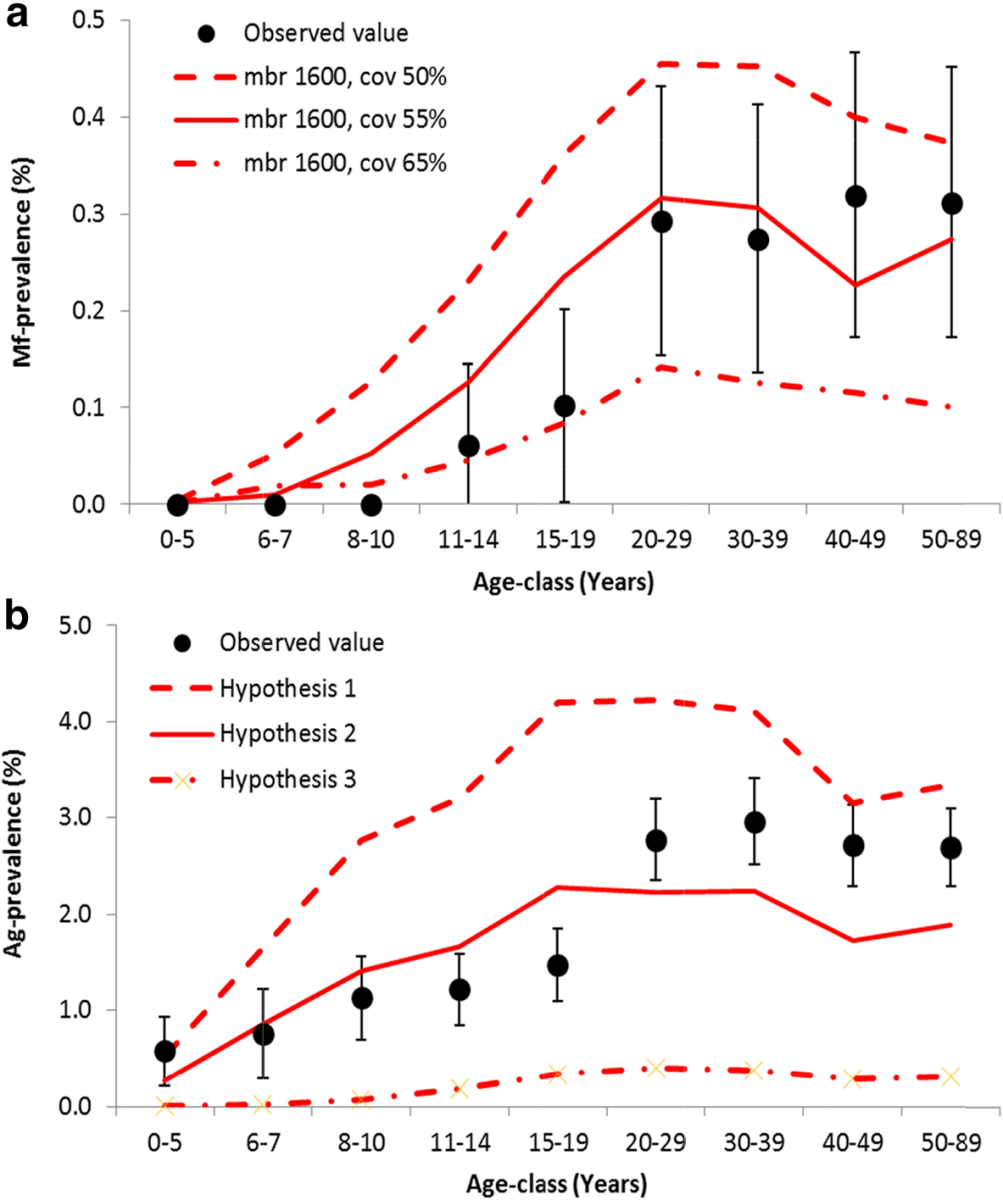
Observed and model predicted age-specific Mf and Ag prevalence post-MDA. Empirical data are from two primary health centres in Thanjavur district, India, where 8 rounds MDA took place Thanjavur (MDA with DEC alone was given in 1997, 1999, 2000, and 2004; MDA with the combination DEC + ALB was given in 2001, 2002, 2003, and 2007). The model predictions show expected post-MDA age-prevalence patterns for a setting with low baseline endemicity (assumed mbr = 1600), with MDA rounds scheduled as in Thanjavur. **a** Visual qualitative comparison of model predictions to age-specific Mf prevalence data, under different assumptions for the achieved coverage per treatment round; **b** Visual qualitative comparison of model predictions to age-specific Ag prevalence data, under different hypotheses for the association between presence of worms and antigenaemia

Figure 2 shows the qualitative comparison of model predictions to observed Mf and Ag prevalence data by age after 8 rounds of MDA (4 with DEC and 4 with DEC + ALB). The age-patterns of Mf-prevalence could not exactly be reproduced, but the absolute level in adults was adequately matched when we assumed 55 % treatment coverage per round (Fig. 2a) and this coverage figure was therefore used for the comparisons shown in Fig. 2b. Figure 2b shows that hypothesis 1 overestimates the Ag prevalence in all age-groups, while hypothesis 3 results in a strong underestimation. Predictions obtained under hypothesis 2 are in the right order of magnitude, although the levels in adults were somewhat underestimated. The overestimation of Ag prevalence in teenagers is balanced by the overestimated Mf prevalence in this age group. Based on the combined results presented in Figs. 1 and 2, we adopt hypothesis 2 for predicting Ag prevalence levels in the remainder of the manuscript.

### Required duration of MDA for eliminating LF

**Fig. 3:**
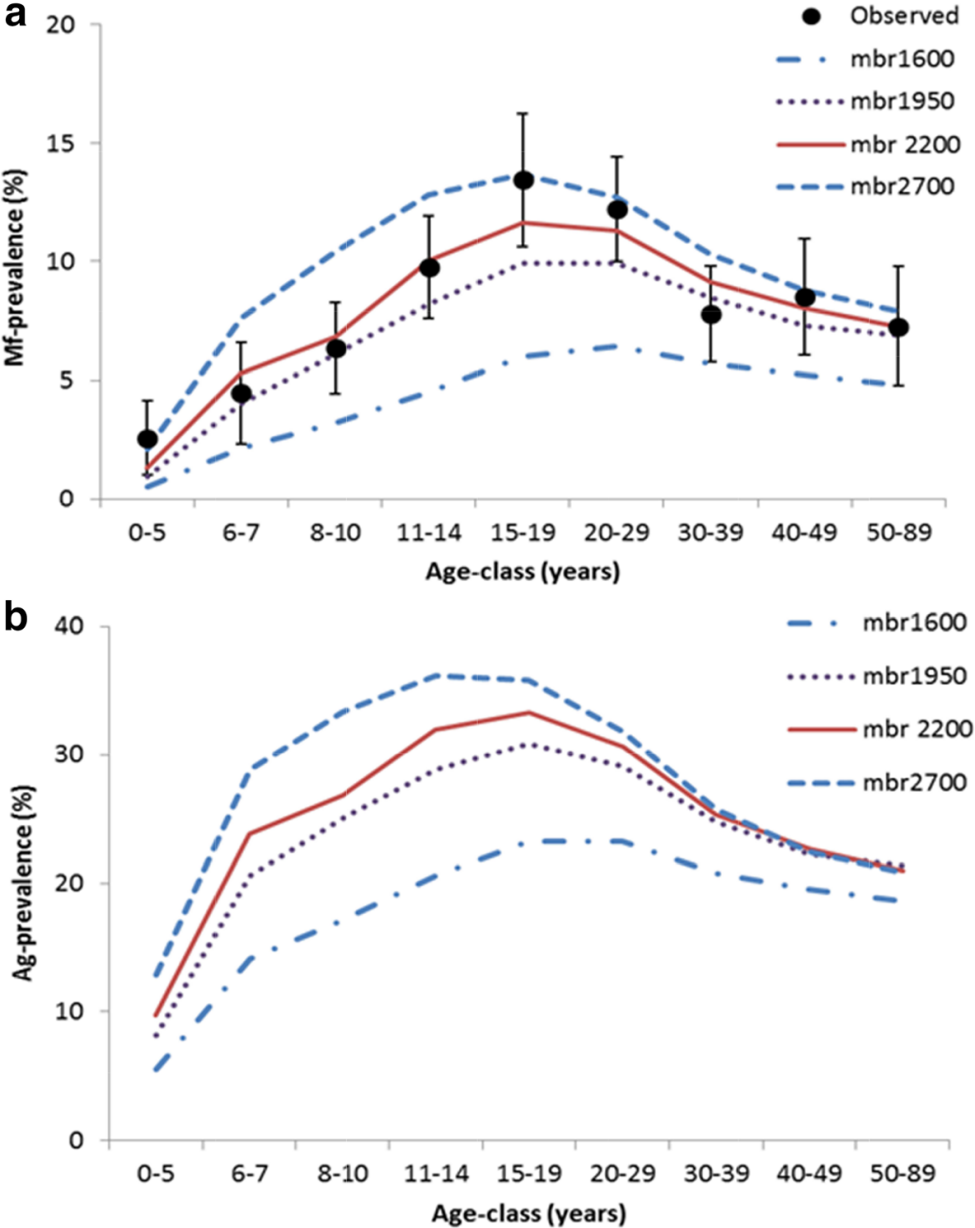
Age patterns of Mf (**a**) and Ag (**b**) prevalence of infection prior to MDA in the four simulated endemic settings. Antigenaemia is assumed to be detectable if at least one male or female worm is present in the host, but the detection rate increases with the number of adult worms (hypothesis 2). The model-predicted pattern of Mf-prevalence for Pondicherry (solid red line) matched well to the observed pattern (dots) from 1981. The predicted Mf prevalence prior to MDA at community level (8.5 %) for Pondicherry was within the range of the observed prevalence (8.6 %; 95 % CI: 7.9–9.4 %), as was the prevalence (5.3 %) in 6–7 year-old children (4.5 %; 95 % CI: 2.3–6.6 %). The model clearly mirrors the observed decline in prevalence in higher age groups (above 30 years)

We considered four epidemiological settings in our simulation experiment, reflecting sites with different mean biting rates. Details about the endemic situation at baseline for different endemic settings are shown in Fig. 3. In all the settings, the mean predicted Mf prevalence increases with age with the maximum peak attained at the age of 20 years, followed by a decline up to the age of 39 years and stabilization at later ages (Fig. 3a). The pattern of age-specific Ag-prevalence is qualitatively similar in all endemic settings (Fig. 3b). As was also shown previously [14], the predicted age-patterns of Mf prevalence match well to observed data for Pondicherry. Table 2 shows the number of annual MDAs needed for achieving ≥ 99 % probability of infection elimination for different endemic settings with varying treatment coverages. In the low endemic setting, the number of MDAs needed (2–4 rounds) is fewer than in settings with intermediate (3–7) and high (4–12) baseline endemicity. The required duration doubles or trebles with decreasing coverage levels for all settings or increasing endemicity: 2–4 rounds of MDA at 80 % coverage to 4–12 rounds with 50 % coverage.

**Table 2:** Number of annual mass treatments required to achieve ≥ 99 % probability of elimination in relation to varying coverage and MDA

Endemic setting (Monthly biting rate)	No. of MDA rounds required at different levels of coverage		
	50 %	65 %	80 %
Low (1600)	4	2	2
Intermediate (1950)	7	4	3
Pondicherry (2200)	8	5	3
High (2700)	12	6	4

### Residual infection prevalence 1-year post-MDA after required treatment duration

**Fig. 4:**
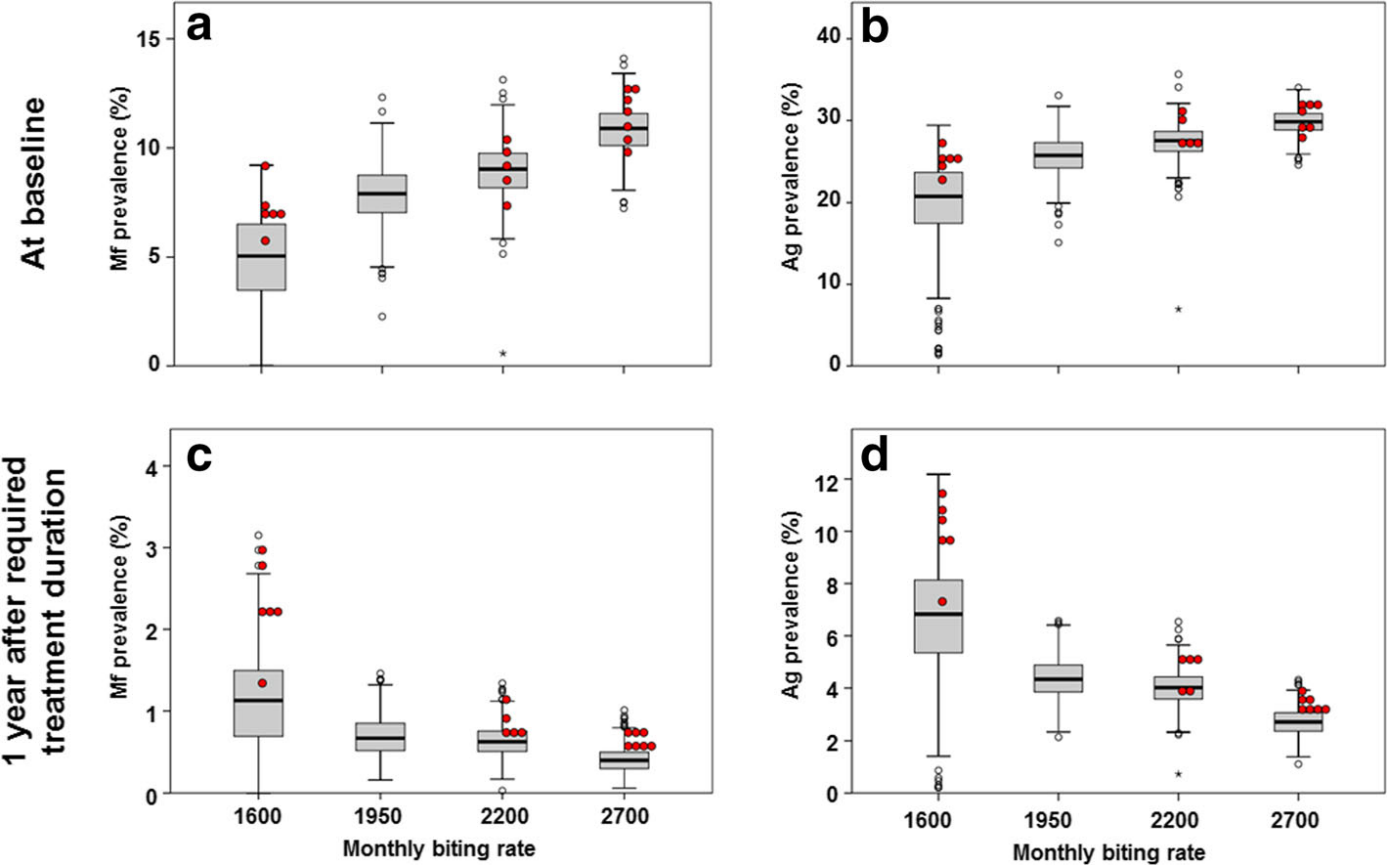
Predicted Mf and Ag prevalence for the population aged 5 years and above, prior to MDA (**a** & **b**) and 1 year after the required treatment duration (**c** & **d**). Antigenaemia is assumed to be detectable if at least one male or female worm is present in the host, but the detection rate increases with the number of adult worms (hypothesis 2). The boxes show the 25th and 75th percentiles of the distribution of the prevalence values and the horizontal line across the box is the median prevalence. The whiskers extend to 1.5 times the height of the box (i.e. the interquartile range, IQR) or, if no case/row has a value in that range, to the minimum or maximum values. If the data are distributed normally, approximately 95 % of the data are expected to lie between the inner fences. Values more than three IQR’s from the end of a box are labelled as extreme, denoted with an asterisk (*). Values more than 1.5 IQR’s but less than 3 IQR’s from the end of the box are labelled as outliers (o). The boxes combine information from the ~99 % runs ending in elimination and the ~1 % runs that did not achieve the target. The red dots indicate the prevalence levels for the few runs that did not result in elimination

**Fig. 5:**
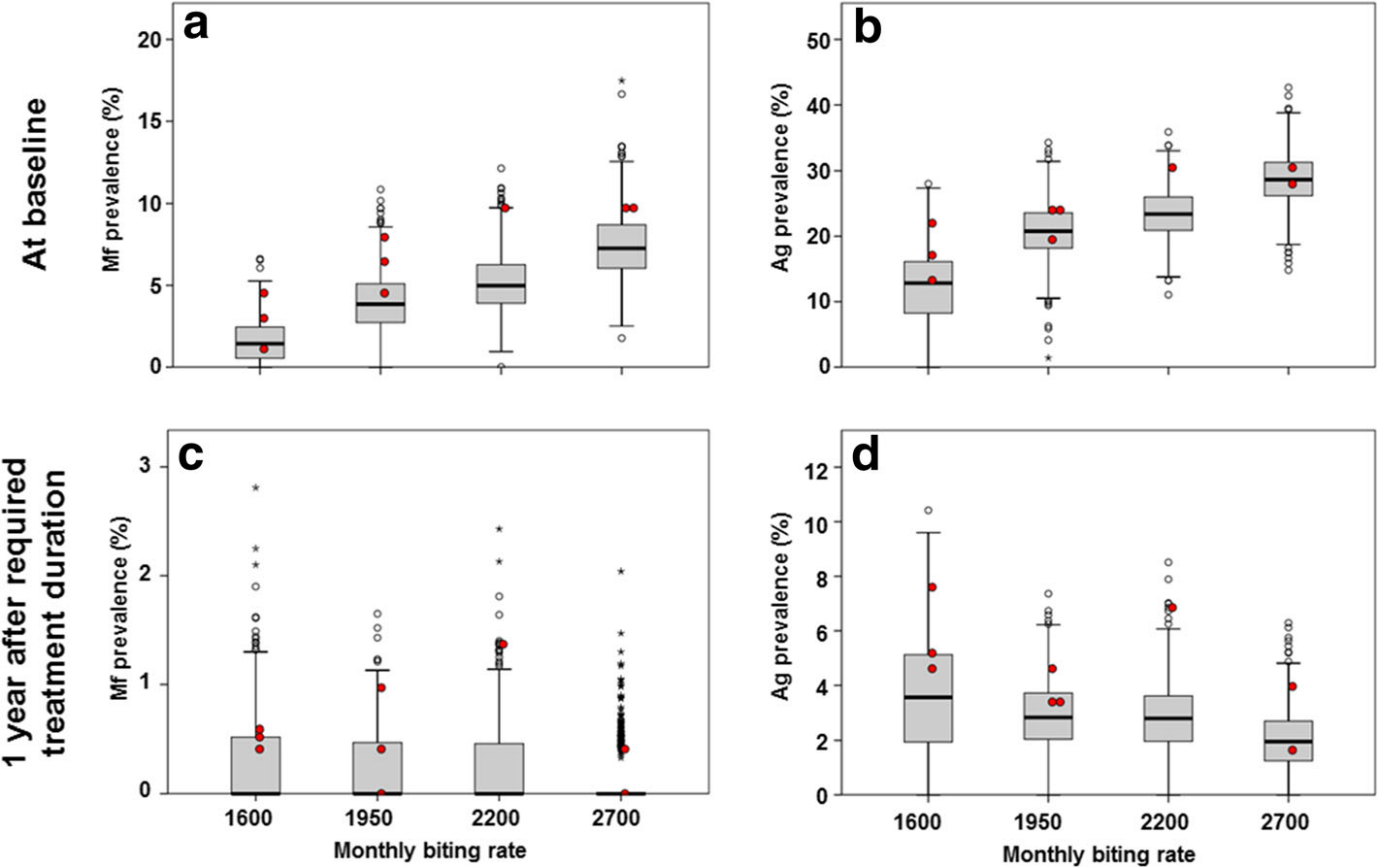
Predicted Mf and Ag prevalence for 6–7 year-old children, prior to MDA (**a** & **b**) and 1 year after the required treatment duration (**c** & **d**). Antigenaemia is assumed to be detectable if at least one male or female worm is present in the host, but the detection rate increases with the number of adult worms (hypothesis 2). See legend to Fig. 4 for additional information regarding the interpretation of the boxplots

Figure 4 summarizes simulation results with respect to the Mf and Ag prevalence in the population aged 5 years and above, prior to MDA and 1 year after the required treatment duration for elimination. We grouped data by setting (mbr), irrespective of treatment scenarios, because the assumed coverage and corresponding treatment duration did not influence residual infection levels 1 year after the last treatment if treatment was continued long enough to achieve elimination, whether achieved by few treatment with high coverage, or more treatment rounds with lower coverage (see Additional file 5: Figure S1 for clustered boxplots by mbr and coverage). Baseline prevalence levels increased with the assumed biting rate, with the median Mf prevalence in the population aged 5 years or older increasing from about 5 % for the lowest mbr to 11 % for the highest value and the Ag prevalence increasing from about 20 to 30 %, respectively. The prediction intervals for different mbr levels show considerable overlap. Whereas the required duration of MDA for achieving ≥ 99 % probability of elimination increased with mbr (see Table 2), the residual infection levels associated with this duration and success probability decreased (panels c and d in Fig. 4, residual infection measured 1 year after the last MDA round of the required number). The median residual Mf prevalence declined from 1.1 % at the lowest mbr to 0.4 % at the highest mbr; similarly, the median residual Ag prevalence declined from 6.8 % at the lowest mbr to 2.8 % at the highest mbr. This pattern is as expected: the probability that a given residual infection level leads to recrudescence increases with the biting rate. In settings with high biting rate, infection prevalence must be reduced to very low levels in order to prevent resurgence, whereas higher residual levels may remain in sites with low biting rates. Qualitatively similar patterns were predicted for infection prevalence in 6–7 year-old children (Fig. 5), except that the median residual Mf prevalence in this age-group after the required number of treatment rounds was zero for all biting rates. The median residual Ag prevalence in this group declined from 3.5 % at the lowest mbr to 2.0 % at the highest.

Red dots in Fig. 4 and Fig. 5 reflect the predicted values for the few runs that failed to achieve elimination (the duration was chosen to result in ≥ 99 % probability of elimination, i.e. up to 1 % of runs did not result in elimination). The residual infection levels were mostly in the upper region of the prediction intervals, as would be expected, but they did not necessarily have the highest values.

### Sensitivity analysis

**Fig. 6:**
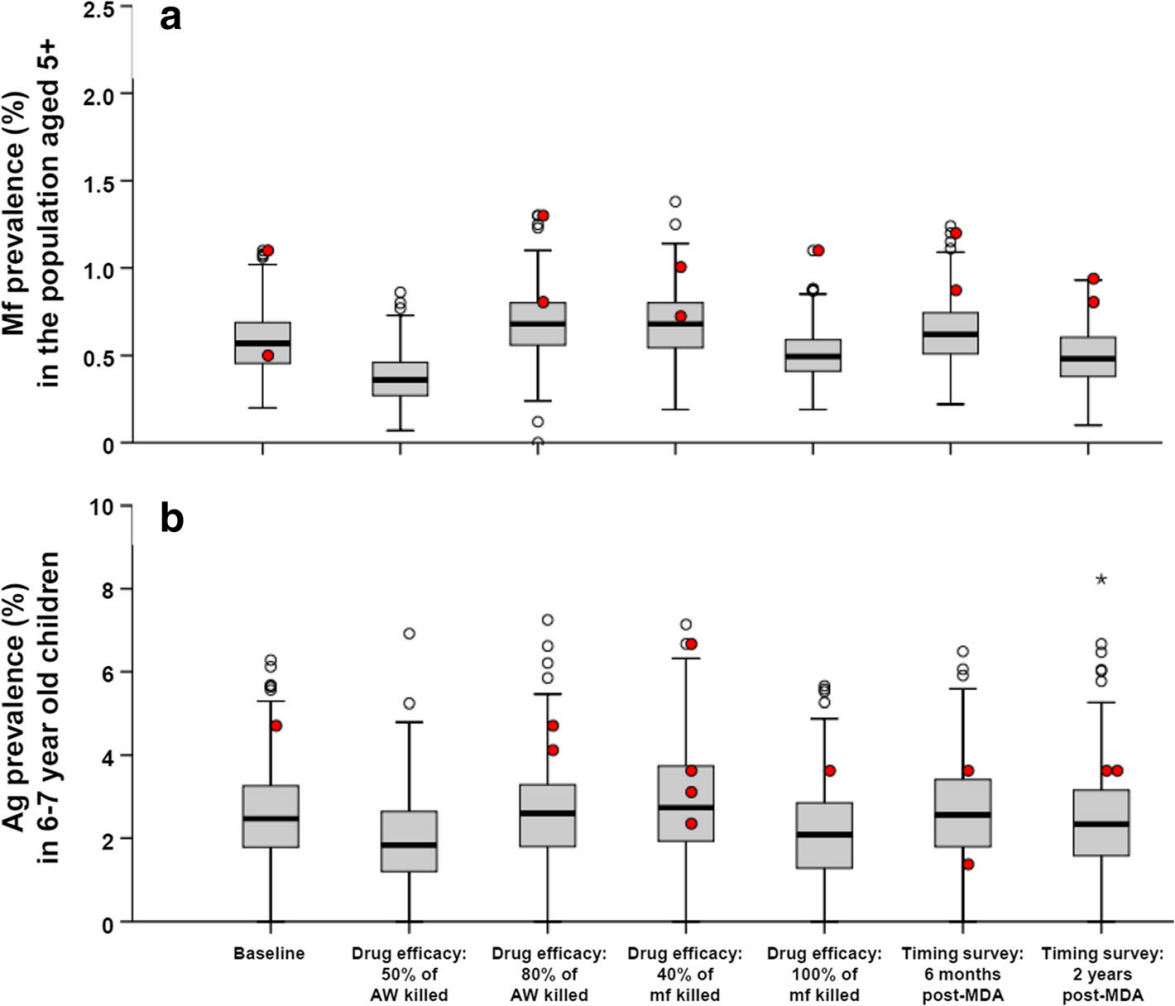
Sensitivity analysis: impact of modified assumptions on the residual Mf (**a**) and Ag (**b**) prevalence that is expected if MDA is continued long enough to achieve elimination with ≥ 99 % probability. See legend to Fig. 4 for additional information regarding the interpretation of the boxplots

In our baseline analysis, 5 rounds of MDA with 65 % would be required to achieve elimination in Pondicherry (Table 2). The required duration changed to 7 and 4 years, when we reduced or increased the percentage of worms killed by a single treatment (50 % or 80 % of worms killed, *versus* 65 % in our baseline analysis). The required duration did not depend on the assumed fraction of Mf killed. The impact of modified assumptions on the residual Mf and Ag prevalence is summarized in Fig. 6. In general, the estimates of residual Mf prevalence post-MDA are more sensitive to the modification of assumptions than estimates of residual Ag prevalence. Residual Mf prevalence levels were lower when we assumed lower adult worm killing per treatment (which in turn was associated with an increase in the duration of mass treatment needed for elimination), higher Mf killing, or longer interval between the last treatment and the epidemiological assessment. The Ag prevalence in 6–7 year-old children changed in the same direction, but the impact was much less pronounced.

## Discussion

GPELF was initiated with a great sense of optimism that yearly mass treatment will lead to elimination of LF. Based on the common assumption that adult worms live for about 5 years, it was thought that 4–6 yearly mass treatment would interrupt transmission if a sufficiently large proportion of the population receives treatment [21] and 2020 was set as target year for global elimination. With this target year coming closer, there is increasing demand for model-based policy support of ongoing elimination programmes, and therefore to further improve available models for lymphatic filariasis. With this vision, three groups hitherto working independently on LF models started to collaborate as members of the Modelling Consortium for Neglected Tropical Diseases [44]. The consortium published a collection of papers in this journal, describing the different models for LF [11, 12] and other NTDs [45]. The current manuscript is part of this collection.

In this study, we used the stochastic microsimulation model LYMFASIM to study how the time to elimination and post-MDA residual infection levels depends on average biting rates (resulting in different baseline endemicity levels) and the achieved coverage in MDA programmes. The model takes account of inter-individual heterogeneities, e.g. in exposure to mosquitoes or compliance with offered treatment, which is known to influence the effectiveness of population-based control measures and the probability of elimination [46].

### Association between presence of infection and antigenaemia

The addition of antigenaemia as new output to the LYMFASIM model is a pre-requisite for broader use of the model to guide country elimination programmes and help refine decision algorithms used to define when MDA can be stopped safely, because antigen detection has become the preferred diagnostic tool for use in TAS [3]. We tested multiple hypotheses about the mechanistic association between adult worm and antigenaemia presence in individuals against data (Figs. 1 and 2). Figure 1 compared the model-predicted association between Mf and Ag prevalence to observed pre-control data collated from the literature. In this selection we observed a higher correlation but otherwise very similar association between the two infection indicators as demonstrated by Cano et al., who based their analysis on a different selection of data [47]. Cano et al. excluded Og4C3-ELISA-based Ag prevalence estimates and included more data from national control programmes (obtained under less well standardized conditions than study data), which may have contributed to the lower correlation between the two infection indicators.

While the antigens derive from adult worms, our comparison of model predictions to data suggests that antigen tests detect only part of the adult worm infection. We tentatively related the detectability of antigenaemia to worm sex, assuming that the contribution of male worms to antigenaemia concentrations may be smaller than that of female worms. The assumption that infections with male worms only remain undetected is perhaps biologically plausible, considering the much smaller size of male worms and presumably much smaller contribution to antigen concentrations in the blood [42], but is not proven by the current analysis. However, through this assumption we capture an important feature, namely the likely association between the host’s adult worm load and antigen detection rate. In our model, the probability of having a single-sex infection declines with increasing worm loads, and therefore the antigen detection rate increases with worm burden (on average antigens are detected in 50 %, 75 %, 87.5 %, … of people carrying 1, 2, 3, … worms, respectively). Qualitatively, this in line with empirical data suggesting imperfect sensitivity by comparing outcomes of different diagnostic tests especially at low worm loads [35, 36, 38, 39].

A limitation of the current implementation of hypothesis 2, is the lack of an explicit sensitivity parameter that could be used to mimic different types of antigen tests. A more flexible and potentially more realistic approach to model antigenaemia mechanistically would involve explicit, quantitative simulation of antigen concentrations in the blood. Assumptions would have to be made regarding the relative contribution of various parasite stages to this concentration and the detection threshold, as has been done for modelling antigen levels for schistosomiasis [48]. Test sensitivity could easily be adjusted for different types of Ag detection test (e.g. for ICT *vs* Alere) by assuming a higher or lower detection threshold. If the detection threshold is in the same order of magnitude as the contribution coming from one female worm, while assuming a much lower contribution from male worms and no contribution from Mf, this quantitative model is comparable to our hypothesis 2.

The comparison of model predicted Ag prevalence levels to data in this study suggest that hypothesis 2 might provide a crude but not unreasonable approximation of the more complex model, whether detection is mediated by worm sex or not. Yet, the hypothesis should be tested more extensively, using different model variants (such as LYMFASIM’s model variant for Africa [16] which does not include a role for acquired immunity and results in considerably higher prevalence levels) and data from other regions. It will be interesting to study whether this hypothesis also accurately predicts Ag prevalence levels as would be found with the new Alere Filariasis Test Strip, which was found to detect more infections [39, 40].

### Required number of treatment rounds

The required number of treatment rounds for achieving elimination was found to increase with baseline endemicity (as proxy for local transmission conditions) and with lower treatment coverage. For instance, in low endemic settings, the number of rounds could be as low as 4 or 2 with a treatment coverage of 50 % or 80 % when compared to that of high settings (12 rounds or 4 rounds). This is in line with our earlier reports, also for other settings [15, 17], and predictions from other models [10, 49]. Estimates of the absolute number of rounds required should be interpreted with care, because they depend on (often unknown) local transmission conditions and uncertain model assumptions [12, 50–52]. Stolk et al. [17] showed how the predicted required duration depends on the assumed efficacy of treatment on adult worms. If the macrofilaricidal effect in reality would be lower than assumed here, the total number of treatment rounds needed to achieve elimination would increase, and vice versa.

### Residual infection prevalence after MDA

TAS with standardized methods are recommended to verify that elimination is achieved in an area under evaluation. When the community Mf prevalence in sentinel and spot-check sites is shown to be below 1 %, TAS is recommended to verify that the average Ag prevalence in 6–7 year-old children is significantly lower than the threshold value of 2 % [3]. We estimated acceptable residual Mf and Ag prevalence levels as the levels associated with 99 % probability of elimination and found that the range of acceptable values extends well above the threshold, suggesting that the proposed threshold is probably safe for most settings to verify for individual communities whether elimination is achieved.

The acceptable residual infection levels were found to decrease with increasing baseline endemicity or biting rate (Figs. 4 and 5). In settings with low baseline endemicity, a higher residual Mf and Ag prevalence may remain post-treatment, because the low biting rate prevents resurgence of transmission. This pattern is theoretically expected [46] and in line with predictions from other models for onchocerciasis and lymphatic filariasis [12, 51, 53]. The levels were independent of the achieved coverage in mass treatment. Although our predictions were for Indian settings only, qualitatively similar patterns are expected regions with other parasite-vector combinations. Considering the negative association between biting rate or baseline endemicity and acceptable residual infection prevalence, it is especially important to confirm that the 2 % threshold is low enough to distinguish between success and failure of elimination programmes even in high-transmission settings. This requires additional simulation work to estimate the probability of elimination in relation to the one-year post-MDA residual infection levels and local transmission conditions.

### Uncertainty in model predictions

Care is required in the interpretation of our results. Uncertainty is inherent in the model-estimated required duration and acceptable post-treatment infection levels and care is required in the interpretation of the presented numbers. Whether or not a certain residual infection level will move the parasite population to extinction or recrudescence, depends on the probability that a worm can mate and successfully reproduce, which in the model is driven by assumptions regarding local transmission intensity, density-dependent processes involved in the transmission, the degree of parasite over-dispersion among hosts in the population, and the interactions of these with the intervention(s) deployed [46, 50, 51, 53, 54]. Our model captures many of the relevant processes, e.g. variation in exposure to mosquito bites, density dependence in transmission from human to vector (limitation in *Culex quinquefasciatus*, facilitation for Anopheles mosquitoes [55–58]) and from vector to human (acquired immunity [14, 59, 60]), and variation between individuals in compliance with treatment [46, 50, 53, 54]. Yet, our qualitative understanding of these processes is still incomplete, which is exemplified by the debated role of acquired immunity [60]. Also, empirical evidence for quantification of these processes is limited. Better qualitative and quantitative understanding of these processes is key to improve the accuracy of critical threshold levels, which will require multidisciplinary approaches, combining knowledge and methods from entomology, biology, epidemiology, mathematics [54].

More work is also needed to understand better how transmission conditions and model parameters can vary between sites and over time, and thus to what extent our findings can be generalized to other settings even if the vector-parasite complex is the same. We simulated hypothetical Indian communities with pre-defined mean exposure and patterns of exposure, under the assumption that model-parameters remain stable over time. While the assumption of geographical and time-stability may seem reasonable for core biological parameters (e.g. related to the parasite lifecycle or host immunity), it is not impossible that some of these parameters change with the hosts’ nutritional or co-infection status. Exposure-related parameters are likely more amenable to variation in space and time. They depend on climatological factors and environmental conditions driving the presence of breeding sites and abundance of mosquitoes, and on the use of personal protection measures (such as window screens, bednets); these parameters in turn are influenced by economic development and likely to change over time. Care is therefore required in the interpretation of long-term predictions and in translating the findings to other specific settings. Other modelling studies indeed confirm that parameter values vary between sites, but parameter values seem to be relatively stable over the typical duration of MDA programmes [12, 52, 61].

### Implications of heterogeneity for elimination programmes

Our results demonstrate how required duration of MDA and post-MDA residual infection levels depend on local transmission conditions and achieved coverage. We did not assess the validity of the full TAS methodology, in which average Ag prevalence in children is assessed in cluster or systematic sample of children from different communities and which also involves repeated assessments several years after stopping MDA. Nevertheless, our results help to illustrate the potential implications of heterogeneity between communities in the evaluation area.

If we assume that all communities within an evaluation area are similar with respect to local transmission conditions (mbr) and operational effectiveness of MDA (number of treatment rounds, coverage and compliance patterns), then the individual boxes in the lower panels of Figs. 4 and 5 show what distribution of residual infection levels would be consistent with ≥ 99 % probability of elimination. However, heterogeneity in transmission conditions and effectiveness of MDA is to be expected within an evaluation area, which might cover over 1000 communities and a population size up to 2 million people. MDA should be continued long enough to ensure that elimination is expected even in communities with highest transmission intensity and lowest coverage. In addition, the critical threshold used to determine whether MDA can stop should be set low enough to ensure successful elimination in the communities with highest transmission intensities. If the threshold is reached in these communities, it is likely that other communities with less intensive transmission have also achieved their threshold (which would be higher, and expected to be achieved in fewer rounds). This implies that, for many communities in that region, MDA would be continued longer than strictly required, resulting in lower residual infection levels than shown in the boxplots.

Rather than ensuring that the average level of residual infection in the area is below a threshold, TAS should be designed to minimize the risk that pockets with unacceptably high residual infection levels remain after cessation of MDA. Ideally, TAS should be targeted at the sites with the most unfavourable conditions for elimination (highest biting rates and lowest coverage). Failure to include such villages may falsely suggest that the critical threshold is achieved everywhere and lead to premature cessation of the elimination programme, local recrudescence of transmission, and eventually reintroduction of infection in surrounding areas. It is therefore crucial to identify these settings, based on community-level predictors of high transmission intensity, poor coverage and poor compliance. Predictors for high transmission intensity could include geographical and environmental factors (e.g. climatic conditions, altitude, vegetation, altitude, population density [47, 62–65], health system and epidemiological features (e.g. bednet coverage [66]), history of mass ivermectin treatment [67, 68]) and socio-economic and sanitary conditions [47]. Possible predictors of poor coverage and compliance include health system and programmatic factors (e.g. drug distribution system, number of drug distributors per population, training of distributors, sensitization of the population for MDA, immunization rate [69–74]), geographic factors (e.g. remoteness, level of urbanization [75–77], and demographic factors (population size, migration, population density [71, 78]).

This study showed that sites with very high biting rates present a particular challenge to elimination programmes. These settings could benefit from vector control, as an adjunct to MDA, which is expected to reduce the duration of control [11, 52]. Adding integrated vector management would help to homogenize the transmission conditions within an evaluation unit within a region and to minimize the risk that pockets with ongoing transmission remain after cessation of MDA. This will, however, increase the overall costs of the programmes but with multiple collateral benefits.

### Prospects for LF elimination by 2020

The Global Programme to Eliminate Lymphatic Filariasis was set up with the aim to eliminate LF globally by 2020. Great progress has been made: as of 2014, 39 of the 73 endemic countries have implemented MDA; 46 countries have completed 5 or more MDA rounds. Of them, 18 countries have already stopped MDA and progressed to the surveillance phase, with 55 countries continuing to require MDA. Eleven countries have yet to start MDA [79]. Although the global programme to eliminate LF has been successful to date, It has been recognized that coverage will have to be scaled up substantially if the 2020 target is to be achieved [80].

In India, LF is endemic in 255 districts from 20 States/Union Territories, with a total of about 600 million people at risk. By 2015, most endemic districts had completed the five annual rounds of mass drug administration (MDA) with the diethylcarbamazine-albendazole drug-combination (DEC + ALB), with good reported coverage. Mf prevalence surveys performed in sentinel and spot check sites suggest that the overall Mf prevalence in endemic areas has been reduced from 1.24 % in 2004 to about 0.3 % in 2013, and about 200 districts have reported overall Mf prevalence levels < 1 % [81]. However, much heterogeneity is to be expected, both in baseline endemicity levels (known to vary between districts and communities [60, 82] and in achieved coverage. Reported coverage seems adequate, but there is great concern about the gap between the number of tablets distributed and the actual ingestion of the drugs [83]. As shown in this paper, both factors influence the required duration of MDA for achieving elimination. Several epidemiological studies confirmed that infection may persist after long-term MDA [84–86], with spatial clustering in hotspots with potential for resurgence of infection. The large size of implementation units implies that there is huge potential for hotspots to remain undetected in pre-TAS and TAS surveys. Better targeting of TAS to sites expected to have high transmission potential or low achieved coverage would help to reduce this risk.

## Conclusions

Our simulation study provided plausible ranges of required post-treatment values for Mf and Ag prevalence, at community level as well as for children of 6–7 year-old, which are associated with ≥ 99 % probability of elimination in Indian settings, where parasite *W. bancrofti* is transmitted by the vector *Cx. quinquefasciatus*. The TAS requires that the Ag prevalence in 6–7 old children is brought below 2 %, and this threshold falls well below the upper level of the range of predicted infection levels associated with ≥ 99 % probability of elimination. The acceptable level of residual Mf prevalence was found to substantially decrease with increasing baseline endemicity. Qualitatively similar patterns are expected in other regions. In practice therefore, the critical threshold should be chosen low enough to also result in elimination in high endemic settings. To ensure the achievement of elimination throughout an evaluation area, TAS should be targeted at the sites with the highest transmission intensity and lowest coverage.

## Additional files

Additional file 1: A zip file, containing instructions, executables and input files for running LYMFASIM. (ZIP 118 kb) Additional file 2: A zip-file, containing the source-code of the LYMFASIM computer program. The contents of this zip archive are licensed under the Creative Commons Attribution-NonCommercial-NoDerivatives 4.0 International License. To view a copy of this license, visit http://creativecommons.org/licenses/by-nc-nd/4.0/or send a letter to Creative Commons, PO Box 1866, Mountain View, CA 94042, USA. By opening Additional file 2, you agree to the aforementioned license. You are free to use and share (copy and redistribute the material in any medium or format) the material contained within Additional File 2 under the following terms: Attribution: You must give appropriate credit, provide a link to the license, and indicate if changes were made. You may do so in any reasonable manner, but not in any way that suggests the licensor endorses you or your use. NonCommercial: You may not use the material for commercial purposes. No Derivatives: If you remix, transform, or build upon the material, you may not distribute the modified material. (ZIP 188 kb) Additional file 3: Mathematical description of the model, including an overview of user-defined input assumptions and parameter values used in the current simulation study. (DOCX 373 kb) Additional file 4: Excel document, summarizing the empirical data on pre-control association between Mf and Ag prevalence that were obtained from literature. (XLSX 26 kb) Additional file 5: Figure S1. For clustered boxplots by mbr and coverage. (PDF 112 kb)

## Abbreviations

Ag: Antigen, antigenaemia
DEC + ALB: Diethylcarbamazine and albendazole
GPELF: Global programme to eliminate lymphatic filariasis
IVM + ALB: Ivermectin and albendazole
LF: Lymphatic filariasis
mbr: Monthly biting rate
MDA: Mass drug administration
Mf: Microfilariae, microfilarial, microfilaraemia
SPSS: Statistical package for social sciences
TAS: Transmission assessment survey

## References

[1] Ottesen EA, Duke BOL, Karam M, Behbehani K (1997). Strategies and tools for the control/elimination of lymphatic filariasis. Bull World Health Organ.

[2] World Health Organization (1997). Elimination of lymphatic filariasis as a public health problem - resolution of the executive board of the WHO (WHA50.29).

[3] World Health Organization (2011). Global Programme to Eliminate Lymphatic filariasis: monitoring and epidemiological assessment of mass drug administration. A manual for national elimination programmes.

[4] WHO Regional Office for South-East Asia (2010). The regional strategic plan for elimination of lymphatic filariasis 2010–2015.

[5] Michael E, Malecela-Lazaro MN, Kabali C, Snow LC, Kazura JW (2006). Mathematical models and lymphatic filariasis control: endpoints and optimal interventions. Trends Parasitol.

[6] Michael E, Malecela-Lazaro MN, Maegga BT, Fischer P, Kazura JW (2006). Mathematical models and lymphatic filariasis control: monitoring and evaluating interventions. Trends Parasitol.

[7] Chu BK, Deming M, Biritwum NK, Bougma WR, Dorkenoo AM, El-Setouhy M (2013). Transmission assessment surveys (TAS) to define endpoints for lymphatic filariasis mass drug administration: a multicenter evaluation. PLoS Negl Trop Dis.

[8] Swaminathan S, Perumal V, Adinarayanan S, Kaliannagounder K, Rengachari R, Purushothaman J (2012). Epidemiological assessment of eight rounds of mass drug administration for lymphatic filariasis in India: implications for monitoring and evaluation. PLoS Negl Trop Dis.

[9] Directorate of National Vector Borne Disease Control Programme. Elimination of lymphatic filariasis, India: 2013–2014. National guidelines for transmission assessment survey (for district & state level health officials). Government of India, Delhi. 2014. http://nvbdcp.gov.in/Doc/TAS-National-Guidelines-2013-14.pdf. Accessed 27 Aug 2016.

[10] Stolk WA, Stone C, de Vlas SJ (2015). Modelling lymphatic filariasis transmission and control: modelling frameworks, lessons learned and future directions. Adv Parasitol.

[11] Irvine MA, Reimer LJ, Njenga SM, Gunawardena S, Kelly-Hope L, Bockarie M (2015). Modelling strategies to break transmission of lymphatic filariasis - aggregation, adherence and vector competence greatly alter elimination. Parasit Vectors.

[12] Singh BK, Michael E (2015). Bayesian calibration of simulation models for supporting management of the elimination of the macroparasitic disease, lymphatic filariasis. Parasit Vectors.

[13] Plaisier AP, Subramanian S, Das PK, Souza W, Lapa T, Furtado AF (1998). The LYMFASIM simulation program for modeling lymphatic filariasis and its control. Methods Inf Med.

[14] Subramanian S, Stolk WA, Ramaiah KD, Plaisier AP, Krishnamoorthy K, Van Oortmarssen GJ (2004). The dynamics of *Wuchereria bancrofti* infection: a model-based analysis of longitudinal data from Pondicherry, India. Parasitology.

[15] Stolk WA, Subramanian S, Oortmarssen GJ, Das PK, Habbema JDF (2003). Prospects for elimination of bancroftian filariasis by mass drug treatment in Pondicherry, India: a simulation study. J Infect Dis.

[16] Stolk WA, de Vlas SJ, Borsboom GJ, Habbema JD (2008). LYMFASIM, a simulation model for predicting the impact of lymphatic filariasis control: quantification for African villages. Parasitology.

[17] Stolk WA, ten Bosch QA, de Vlas SJ, Fischer PU, Weil GJ, Goldman AS (2013). Modeling the impact and costs of semiannual mass drug administration for accelerated elimination of lymphatic filariasis. PLoS Negl Trop Dis.

[18] Subramanian S, Pani SP, Ravi R, Krishnamoorthy K, Das PK (2008). Mathematical models for lymphatic filariasis transmission and control: Challenges and prospects. Parasit Vectors.

[19] Habbema JDF, De Vlas SJ, Plaisier AP, Van Oortmarssen GJ (1996). The microsimulation approach to epidemiologic modeling of helminthic infections, with special reference to schistosomiasis. Am J Trop Med Hyg.

[20] De Kraker MEA, Stolk WA, Van Oortmarssen GJ, Habbema JDF (2006). Model-based analysis of trial data: microfilaria and worm-productivity loss after diethylcarbamazine–albendazole or ivermectin–albendazole combination therapy against *Wuchereria bancrofti*. Trop Med Int Health.

[21] Ottesen EA, Ismail MM, Horton J (1999). The role of albendazole in programmes to eliminate lymphatic filariasis. Parasitol Today.

[22] Kshirsagar NA, Gogtay NJ, Garg BS, Deshmukh PR, Rajgor DD, Kadam VS (2004). Safety, tolerability, efficacy and plasma concentrations of diethylcarbamazine and albendazole co-administration in a field study in an area endemic for lymphatic filariasis in India. Trans R Soc Trop Med Hyg.

[23] El Setouhy M, Ramzy RMR, Ahmed ES, Kandil AM, Hussain O, Farid HA (2004). A randomized clinical trial comparing single- and multi-dose combination therapy with diethylcarbamazine and albendazole for treatment of bancroftian filariasis. Am J Trop Med Hyg.

[24] Plaisier AP, Stolk WA, van Oortmarssen GJ, Habbema JD (2000). Effectiveness of annual ivermectin treatment for *Wuchereria bancrofti* infection. Parasitol Today.

[25] Vanamail P, Ramaiah KD, Subramanian S, Pani SP, Yuvaraj J, Das PK (2005). Pattern of community compliance with spaced, single-dose, mass administrations of diethylcarbamazine or ivermectin, for the elimination of lymphatic filariasis from rural areas of southern India. Ann Trop Med Parasitol.

[26] Weil GJ, Lammie PJ, Weiss N (1997). The ICT filariasis test: a rapid-format antigen test for diagnosis of bancroftian filariasis. Parasitol Today.

[27] Weil GJ, Ramzy RM. Diagnostic tools for filariasis elimination programs. Trends Parasitol. 2007;23:78-82. 10.1016/j.pt.2006.12.001 17174604

[28] Chanteau S, Moulia-Pelat JP, Glaziou P, Nguyen NL, Luquiaud P, Plichart C (1994). Og4C3 circulating antigen: a marker of infection and adult worm burden in *Wuchereria bancrofti* filariasis. J Infect Dis.

[29] Weil GJ, Jain DC, Santhanam S, Malhotra A, Kumar H, Sethumadhavan KV (1987). A monoclonal antibody-based enzyme immunoassay for detecting parasite antigenemia in bancroftian filariasis. J Infect Dis.

[30] Nicolas L (1997). New tools for diagnosis and monitoring of bancroftian filariasis parasitism: the Polynesian experience. Parasitol Today.

[31] Weil GJ, Ramzy RM, Chandrashekar R, Gad AM, Lowrie RC, Faris R (1996). Parasite antigenemia without microfilaremia in bancroftian filariasis. Am J Trop Med Hyg.

[32] Sunish IP, Rajendran R, Satyanarayana K, Munirathinam A, Gajanana A (2001). Immunochromatographic test (ICT) for estimation of true prevalence of bancroftian filariasis in an endemic area in southern India. Trans R Soc Trop Med Hyg.

[33] Pani SP, Hoti SL, Vanamail P, Das LK (2004). Comparison of an immunochromatographic card test with night blood smear examination for detection of *Wuchereria bancrofti* microfilaria carriers. Natl Med J India.

[34] Das D, Kumar S, Sahoo PK, Dash AP (2005). A survey of bancroftian filariasis for microfilariae & circulating antigenaemia in two villages of Madhya Pradesh. Indian J Med Res.

[35] Dreyer G, Lins R, Noroes J, Rizzo JA, Figueredo-Silva J (2008). Sensitivity of the Immunochromatographic card test relative to detection of adult *Wuchereria bancrofti* worms by ultrasound. Am J Trop Med Hyg.

[36] Nguyen NL, Plichart C, Esterre P (1999). Assessment of immunochromatographic test for rapid lymphatic filariasis diagnosis. Parasite.

[37] Gass K, de Beau Rochars MV, Boakye D, Bradley M, Fischer PU, Gyapong J (2012). A multicenter evaluation of diagnostic tools to define endpoints for programs to eliminate bancroftian filariasis. PLoS Negl Trop Dis.

[38] Gounoue-Kamkumo R, Nana-Djeunga HC, Bopda J, Akame J, Tarini A, Kamgno J (2015). Loss of sensitivity of immunochromatographic test (ICT) for lymphatic filariasis diagnosis in low prevalence settings: consequence in the monitoring and evaluation procedures. BMC Infect Dis.

[39] Weil GJ, Curtis KC, Fakoli L, Fischer K, Gankpala L, Lammie PJ (2013). Laboratory and field evaluation of a new rapid test for detecting *Wuchereria bancrofti* antigen in human blood. Am J Trop Med Hyg.

[40] Yahathugoda TC, Supali T, Rao RU, Djuardi Y, Stefani D, Pical F (2015). A comparison of two tests for filarial antigenemia in areas in Sri Lanka and Indonesia with low-level persistence of lymphatic filariasis following mass drug administration. Parasit Vectors.

[41] Weil GJ, Liftis F (1987). Identification and partial characterization of a parasite antigen in sera from humans infected with *Wuchereria bancrofti*. J Immunol.

[42] Centers for Disease Control and Prevention (CDC). DPDx - Laboratory identification of parasitic diseases of public health concern. Lymphatic filariasis. Atlanta: Centers for Disease Control and Prevention; http://www.cdc.gov/dpdx/lymphaticFilariasis/. Accessed 27 Aug 2016.

[43] Stolk WA, De Vlas SJ, Habbema JDF (2005). Anti-*Wolbachia* treatment for lymphatic filariasis. Lancet.

[44] NTD Modelling Consortium. http://www.ntdmodelling.org/. Accessed 27 Aug 2016.

[45] Hollingsworth TD, Adams ER, Anderson RM, Atkins K, Bartsch S, Basanez MG (2015). Quantitative analyses and modelling to support achievement of the 2020 goals for nine neglected tropical diseases. Parasit Vectors.

[46] Duerr HP, Dietz K, Eichner M (2005). Determinants of the eradicability of filarial infections: a conceptual approach. Trends Parasitol.

[47] Cano J, Rebollo MP, Golding N, Pullan RL, Crellen T, Soler A (2014). The global distribution and transmission limits of lymphatic filariasis: past and present. Parasit Vectors.

[48] Polman K, de Vlas SJ, Gryseels B, Deelder AM (2000). Relating serum circulating anodic antigens to faecal egg counts in *Schistosoma mansoni* infections: a modelling approach. Parasitology.

[49] Michael E, Malecela-Lazaro MN, Simonsen PE, Pedersen EM, Barker G, Kumar A (2004). Mathematical modelling and the control of lymphatic filariasis. Lancet Infect Dis.

[50] Gambhir M, Michael E (2008). Complex ecological dynamics and eradicability of the vector borne macroparasitic disease, lymphatic filariasis. PLoS One.

[51] Gambhir M, Bockarie M, Tisch D, Kazura J, Remais J, Spear R (2010). Geographic and ecologic heterogeneity in elimination thresholds for the major vector-borne helminthic disease, lymphatic filariasis. BMC Biol.

[52] Michael E, Singh BK (2016). Heterogeneous dynamics, robustness/fragility trade-offs, and the eradication of the macroparasitic disease, lymphatic filariasis. BMC Med.

[53] Duerr HP, Raddatz G, Eichner M (2011). Control of onchocerciasis in Africa: threshold shifts, breakpoints and rules for elimination. Int J Parasitol.

[54] Basáñez MG, McCarthy JS, French MD, Yang GJ, Walker M, Gambhir M (2012). A research agenda for helminth diseases of humans: modelling for control and elimination. PLoS Negl Trop Dis.

[55] Subramanian S, Krishnamoorthy K, Ramaiah KD, Habbema JDF, Das PK, Plaisier AP (1998). The relationship between microfilarial load in the human host and uptake and development of *Wuchereria bancrofti* microfilariae by *Culex quinquefasciatus*: a study under natural conditions. Parasitology.

[56] Pichon G (2002). Limitation and facilitation in the vectors and other aspects of the dynamics of filarial transmission: the need for vector control against *Anopheles*-transmitted filariasis. Ann Trop Med Parasitol.

[57] Stolk WA, Van Oortmarssen GJ, Subramanian S, Das PK, Borsboom GJJM, Habbema JDF (2004). Assessing density dependence in the transmission of lymphatic filariasis: uptake and development of *Wuchereria bancrofti* microfilariae in the vector mosquitoes. Med Vet Entomol.

[58] Snow LC, Bockarie MJ, Michael E (2006). Transmission dynamics of lymphatic filariasis: vector-specific density dependence in the development of *Wuchereria bancrofti* infective larvae in mosquitoes. Med Vet Entomol.

[59] Michael E, Simonsen PE, Malecela M, Jaoko WG, Pedersen EM, Mukoko D (2001). Transmission intensity and the immunoepidemiology of bancroftian filariasis in East Africa. Parasite Immunol.

[60] Stolk WA, Ramaiah KD, Van Oortmarssen GJ, Das PK, Habbema JDF, De Vlas SJ (2004). Meta-analysis of age-prevalence patterns in lymphatic filariasis: no decline in microfilaraemia prevalence in older age groups as predicted by models with acquired immunity. Parasitology.

[61] Singh BK, Bockarie MJ, Gambhir M, Siba PM, Tisch DJ, Kazura J (2013). Sequential modelling of the effects of mass drug treatments on anopheline-mediated lymphatic filariasis infection in Papua New Guinea. PLoS One.

[62] Lindsay SW, Thomas CJ (2000). Mapping and estimating the population at risk from lymphatic filariasis in Africa [In Process Citation]. Trans R Soc Trop Med Hyg.

[63] Sabesan S, Raju HK, Srividya A, Das PK (2006). Delimitation of lymphatic filariasis transmission risk areas: a geo-environmental approach. Filaria J.

[64] Slater H, Michael E (2012). Predicting the current and future potential distributions of lymphatic filariasis in Africa using maximum entropy ecological niche modelling. PLoS One.

[65] Stanton MC, Molyneux DH, Kyelem D, Bougma RW, Koudou BG, Kelly-Hope LA (2013). Baseline drivers of lymphatic filariasis in Burkina Faso. Geospat Health.

[66] Okorie PN, Ademowo GO, Saka Y, Davies E, Okoronkwo C, Bockarie MJ (2013). Lymphatic filariasis in Nigeria; micro-stratification overlap mapping (MOM) as a prerequisite for cost-effective resource utilization in control and surveillance. PLoS Negl Trop Dis.

[67] Richards FO, Eigege A, Pam D, Kal A, Lenhart A, Oneyka JO (2005). Mass ivermectin treatment for onchocerciasis: lack of evidence for collateral impact on transmission of Wuchereria bancrofti in areas of co-endemicity. Filaria J.

[68] Koroma JB, Sesay S, Sonnie M, Hodges MH, Sahr F, Zhang Y (2013). Impact of three rounds of mass drug administration on lymphatic filariasis in areas previously treated for onchocerciasis in Sierra Leone. PLoS Negl Trop Dis.

[69] World Health Organization (1996). Community directed treatment with ivermectin: report of a multi-country study.

[70] Amazigo U, Okeibunor J, Matovu V, Zoure H, Bump J, Seketeli A (2007). Performance of predictors: evaluating sustainability in community-directed treatment projects of the African programme for onchocerciasis control. Soc Sci Med.

[71] Brieger WR, Otusanya SA, Oke GA, Oshiname FO, Adeniyi JD (2002). Factors associated with coverage in community-directed treatment with ivermectin for onchocerciasis control in Oyo State, Nigeria. Trop Med Int Health.

[72] Krentel A, Fischer PU, Weil GJ (2013). A review of factors that influence individual compliance with mass drug administration for elimination of lymphatic filariasis. PLoS Negl Trop Dis.

[73] Hussain MA, Sitha AK, Swain S, Kadam S, Pati S (2014). Mass drug administration for lymphatic filariasis elimination in a coastal state of India: a study on barriers to coverage and compliance. Infect Dis Poverty.

[74] Kisoka WJ, Tersbol BP, Meyrowitsch DW, Simonsen PE, Mushi DL. Community Members’ Perceptions of Mass Drug Administration for Control of Lymphatic Filariasis in Rural and Urban Tanzania. J Biosoc Sci. 2016;48:94-112. 10.1017/S0021932015000024 PMC4668335 25790081

[75] Gyapong M, Gyapong JO, Owusu-Banahene G (2001). Community-directed treatment: the way forward to eliminating lymphatic filariasis as a public-health problem in Ghana. Ann Trop Med Parasitol.

[76] Hopkins AD (1998). Mectizan delivery systems and cost recovery in the Central African Republic. Ann Trop Med Parasitol.

[77] Ghosh S, Samanta A, Kole S (2013). Mass drug administration for elimination of lymphatic filariasis: Recent experiences from a district of West Bengal, India. Trop Parasitol.

[78] Bhullar N, Maikere J (2010). Challenges in mass drug administration for treating lymphatic filariasis in Papua, Indonesia. Parasit Vectors.

[79] Global programme to eliminate lymphatic filariasis: progress report, 2014. Wkly Epidemiol Rec. 2015;90(38):489–504. 26387149

[80] Stone CM, Kastner R, Steinmann P, Chitnis N, Tanner M, Tediosi F (2016). Modelling the health impact and cost-effectiveness of lymphatic filariasis eradication under varying levels of mass drug administration scale-up and geographic coverage. BMJ Global Health.

[81] Guidelines on elimination of lymphatic filariasis, India. Delhi: Directorate of National Vector Borne Disease Control Programme, Directorate General of Health Services, Ministry of Health & Family Welfare, Government of India. 2009.

[82] Sabesan S, Raju KH, Subramanian S, Srivastava PK, Jambulingam P (2013). Lymphatic filariasis transmission risk map of India, based on a geo-environmental risk model. Vector Borne Zoonotic Dis.

[83] Babu BV, Babu GR (2014). Coverage of, and compliance with, mass drug administration under the programme to eliminate lymphatic filariasis in India: a systematic review. Trans R Soc Trop Med Hyg.

[84] Shriram AN, Krishnamoorthy K, Sivan A, Saha BP, Kumaraswami V, Vijayachari P (2014). Impact of MDA and the prospects of elimination of the lone focus of diurnally sub periodic lymphatic filariasis in Nicobar Islands, India. Acta Trop.

[85] Sunish IP, Munirathinam A, Kalimuthu M, Ashok Kumar V, Tyagi BK (2014). Persistence of lymphatic filarial infection in the paediatric population of rural community, after six rounds of annual mass drug administrations. J Trop Pediatr.

[86] Sunish IP, Kalimuthu M, Rajendran R, Munirathinam A, Ashok Kumar V, Nagaraj J (2015). Decline in lymphatic filariasis transmission with annual mass drug administration using DEC with and without albendazole over a 10 year period in India. Parasitol Int.

[87] Stolk WA, van Oortmarssen GJ, Pani SP, de Vlas SJ, Subramanian S, Das PK (2005). Effects of ivermectin and diethylcarbamazine on microfilariae and overall microfilaria production in bancroftian filariasis. Am J Trop Med Hyg.

