# Supplementary material for: Mathematical modelling of lymphatic filariasis elimination programmes in India: required duration of mass drug administration and post-treatment level of infection indicators
Source: Parasit Vectors. 2016 Sep 13;9(1):501. doi: 10.1186/s13071-016-1768-y (PMC5022201; doi:10.1186/s13071-016-1768-y)
Supplement: Additional file 3: — Mathematical description of the model, including an overview of user-defined input assumptions and parameter values used in the current simulation study. (DOCX 373 kb) [file 13071_2016_1768_MOESM3_ESM.docx]

**The LYMFASIM simulation model – mathematical description and parameter values**

This document is a supplement to the following manuscript:

Jambulingam P, Subramanian S, de Vlas SJ, Vinubala C, Stolk WA. Mathematical modelling of lymphatic filariasis elimination programs in India: required duration of mass drug administration and post-treatment level of infection indicators. Parasites and Vectors (2016)

Contents

[1 Background 3](#_Toc449106747)

[1.1 Development 3](#_Toc449106748)

[1.2 Recommended reading 3](#_Toc449106749)

[1.3 Modelling approach 4](#_Toc449106750)

[1.4 Software 4](#_Toc449106751)

[2 Formal description of LYMFASIM 5](#_Toc449106752)

[2.1 Human demography 5](#_Toc449106753)

[2.2 Transmission of infection 5](#_Toc449106754)

[2.3 Mass treatment coverage and compliance 10](#_Toc449106755)

[2.4 Vector control 12](#_Toc449106756)

[2.5 Surveys 12](#_Toc449106757)

[2.6 Running the model 12](#_Toc449106758)

[3 LYMFASIM input: Probability distributions, functions and parameter values 13](#_Toc449106759)

[4 References 17](#_Toc449106760)

# Background

## Development

The LYMFASIM modelling framework for simulating transmission and control of lymphatic filariasis has been developed by scientists based at Erasmus MC (Rotterdam, the Netherlands), in very close collaboration with the Vector Control Research Centre, Pondicherry, India (Dr. P.K. Das, dr. S. Subramanian), and with additional inputs from scientists based at the Danish Bilharziasis Laboratory, Copenhagen, Denmark (Dr. P.E. Simonsen, Dr. D.W. Meyrowitsch) and Centro de Pesquisas Aggeu Magalhães, Recife, Brazil. (Dr. W. Souza, dr. A. F. Furtado, dr. G. Dreyer).

The model has been developed with financial support from the UNDP/World Bank/ WHO Special Programme for Research and Training in Tropical Diseases (TDR).

Erasmus MC has developed similar models for other diseases, including onchocerciasis (ONCHOSIM [[1](#_ENREF_1)]), schistosomiasis (SCHISTOSIM [[2](#_ENREF_2)]), and most recently also soil-transmitted helminthiasis [[3](#_ENREF_3)].

## Recommended reading

A complete description of the model is given in:

- Plaisier AP, Subramanian S, Das PK, Souza W, Lapa T, Furtado AF, Van der Ploeg CPB, Habbema JDF, Van Oortmarssen GJ. The LYMFASIM simulation program for modeling lymphatic filariasis and its control. *Methods of Information in Medicine*, 1998. 37: p. 97-108.

Quantification of parameters for different endemic regions is described in:

- Subramanian S, Stolk WA, Ramaiah KD, Plaisier AP, Krishnamoorthy K, Van Oortmarssen GJ et al. The dynamics of *Wuchereria bancrofti* infection: a model-based analysis of longitudinal data from Pondicherry, India. Parasitology. 2004;128(Pt 5):467-82.
- Stolk WA, de Vlas SJ, Borsboom GJ, Habbema JD. LYMFASIM, a simulation model for predicting the impact of lymphatic filariasis control: quantification for African villages. Parasitology. 2008;135(13):1583-98.

For information on microsimulation in general and similar models, we refer to:

- Habbema JDF, De Vlas S J, Plaisier AP, Van Oortmarssen G J. The microsimulation approach to epidemiologic modeling of helminthic infections, with special reference to schistosomiasis. Am J Trop Med Hyg, 1996. 55(5 Suppl): p. 165-9.
- Plaisier AP, Van Oortmarssen GJ, Habbema JDF, Remme J, and Alley ES. ONCHOSIM: a model and computer simulation program for the transmission and control of onchocerciasis. Computer Methods and Programs in Biomedicine 1990;31:43-56.

Application of the LYMFASIM model include

- Stolk WA, Subramanian S, Oortmarssen GJ, Das PK, Habbema JDF. Prospects for elimination of bancroftian filariasis by mass drug treatment in Pondicherry, India: a simulation study. J Infect Dis. 2003;188(9):1371-81.
- Subramanian S, Stolk WA, Ramaiah KD, Plaisier AP, Krishnamoorthy K, Van Oortmarssen GJ et al. The dynamics of *Wuchereria bancrofti* infection: a model-based analysis of longitudinal data from Pondicherry, India. Parasitology. 2004;128(Pt 5):467-82.
- Subramanian S. Modelling lymphatic filariasis transmission and control (PhD thesis). Department of Public Health, Erasmus MC. Rotterdam: Erasmus University Rotterdam; 2004.
- Stolk WA, De Vlas SJ, Habbema JDF. Anti-*Wolbachia* treatment for lymphatic filariasis. Lancet. 2005;365(9477):2067-8.
- Stolk WA, de Vlas SJ, Borsboom GJ, Habbema JD. LYMFASIM, a simulation model for predicting the impact of lymphatic filariasis control: quantification for African villages. Parasitology. 2008;135(13):1583-98.
- Stolk WA, ten Bosch QA, de Vlas SJ, Fischer PU, Weil GJ, Goldman AS. Modeling the impact and costs of semiannual mass drug administration for accelerated elimination of lymphatic filariasis. PLoS Negl Trop Dis. 2013;7(1):e1984.

Comparison with other models for lymphatic filariasis:

- Stolk WA, Stone C, de Vlas SJ. Modelling lymphatic filariasis transmission and control: modelling frameworks, lessons learned and future directions. Adv Parasitol. 2015;87:249-91.

## Modelling approach

LYMFASIM is a stochastic computer simulation model for lymphatic filariasis transmission and control in human populations. LYMFASIM simulates the life histories of individual human individuals (together forming a dynamics human population) and individuals parasites, and their transmission from person to person by a vector population. The model can be used to evaluate the effects of different control strategies, such as vector control and chemotherapy. LYMFASIM combines two simulation techniques; stochastic microsimulation is used to calculate the life events of individual persons and their inhabitant parasites [[4](#_ENREF_4)], while the dynamics of infective material in the vector population is simulated deterministically.

## Software

LYMFASIM was implemented in a computer programme, coded in C++. The program source and executables are provided separately with instructions.

# Formal description of LYMFASIM

The model description presented here is based on previously published descriptions [[5-7](#_ENREF_5)].

## Human demography

Each simulations starts with a predefined initial population with specified size and age distribution, typically thought of as representing the population of a single community at a certain timepoint. The population composition changes over time due to processes of birth and death. The human population dynamics is governed by birth and death processes. The probability to die at a certain age *a* is defined by a life table. The cumulative death rate for intermediate ages is obtained by linear interpolation.

The expected number of births (per year) at a given moment *t* is given by:

$R_{b}\left( t \right)=\sum_{a=1}^{n_{a}} N_{f}\left( a,t \right)\cdot r_{b}\left( a \right)$ (1)

with:

$N_{f}\left( a,t \right)$ number of women in age group *a* at time *t*

$r_{b}\left( a \right)$ annual birth rate in age-group *a*.

$n_{a}$ number of age-groups considered.

Each month, $R_{b}\left( t \right)$ is adapted according to the number of women and their age-distribution.

Immigration and emigration are not considered in the LYMFASIM model.

The individual-based structure allows that heterogeneities within the human and parasite population are taken account. The characteristics of individuals are determined by chance and stochastic processes partly determine an individual’s infection load.The models keeps track of the life histories of each human individual, focussing on the acquisition and loss of adult worms, microfilaria density and participation in mass treatment (further explained below).

## Transmission of infection

Figure 1 show the graphical representation of the processes regulating transmission in LYMFASIM. In this figure, *M_i_* is the number of adult worm per individual, *m_i_* the number of microfilariae (scaled to the 20 μL blood volume normally used for diagnosis), and $\bar{L3}$ the average number of L3 larvae present in biting moquitoes. Other symbols are described below.

Figure 1. Schematic representation of transmission processes in LYMFASIM. The shaded boxes are entomological variables; these variables do not vary between individuals. The unshaded boxes are human variables; the index *i* indicates that the value of a parameter of variable varies between human individuals. Along the arrows, the symbols of relevant parameters are shown. Immune regulation is optional in LYMFASIM, with two variants of immunity (anti-L3 and anti-fecundity).

A key-variable in the model is the force-of-infection, *foi*, defined as the number of new immature parasites (i.e., those that survived the larval stage) per month. This *foi* will vary over time and between individuals. For individual *i* at time *t*, it is calculated as follows:

$foi_{i}(t)=mtp_{i}(t)\times sr\times[1-Rl_{i}(t)]$ (2)

The variable *Rl* describes the level of immunity to L3-larvae and may vary between 0 (no immunity) and 1 (full immunity, no L3-larva will survive). Its calculation will be presented below. The parameter *sr* describes the chance that an inoculated L3 larva will survive and reach the stage of immature worm in the absence of immunity ('success-ratio'). Finally, the variable *mtp* is the monthly transmission potential, which follows from:

$mtp_{i}(t)=mbr(t)\times\overline{L3}(t)\times E_{i}(t)$ (3)

with *mbr(t)* quantifying the monthly biting rate (no. of mosquito-bites per month) for an average adult person, $\overline{L3}(t)$being the average number of infective larvae released per vector-bite and *E_i_(t)* quantifying a person's relative exposure. All these variables are time-dependent. The relative exposure of a person has two components: an age-sex dependent component (described by the function *Ea_i_(a(t),s)*) and a random component ('exposure index', *Ei*):

$E_{i}(t)=Ea_{i}(a_{i}(t),s_{i})\times Ei_{i}$ (4)

In many endemic areas the prevalence and intensity of infection among males is higher than among females [[8](#_ENREF_8)], which could reflect sex-differences in exposure. Also in Pondicherry males have the highest prevalence, although the difference between the sexes is only significant for the age-range 20-34 years [[9](#_ENREF_9)]. However, in order to reduce the complexity of the model, in this we assume an equal exposure between the sexes. Possible sex-differences are assumed to be included in random exposure variation (*E_i_*). The age dependent component is described as follows: at birth a person has an exposure of *E_0_*, thereafter it increases linearly to reach a maximum of 1.0 at the age of *a_max_*. The exposure index (random component) is assumed to be a life-long property of a person. Its value is randomly selected from a gamma probability distribution with mean = 1 and shape-parameter *α_E_*. This gamma-distribution allows for persons with low or very low relative exposure.

The average number of infective larvae released per mosquito-bite ($\overline{L3}(t)$) is calculated as follows:

$\overline{L3}(t)=v\times\frac{\sum_{i=1}^{N(t)} E_{i}(t)\times L3_{i}(t)}{\sum_{i=1}^{N(t)} E_{i}(t)}$ (5)

In this expression *N* is the total number of individuals in the human population. *L3_i_* is the average number of L3-larvae resulting from a bloodmeal on person *i* given survival of the mosquito between the uptake of microfilariae and the development to the L3-stage. The linear factor *v* (<1) combines such factors like the probability that an L3-larva will be released, the fraction of potentially infectious mosquitoes (i.e., those that had a bloodmeal before), and survival of the mosquitoes under field conditions.

Based on experimental data, the relation between the human Mf density *m* (as measured by the number of Mf in a blood-smear of 20 μl) and the number of L3 that will develop in feeding mosquitoes (given survival of the mosquitoes) is adequately described by the following hyperbolic function [[10](#_ENREF_10)]:

$L3_{i}(t)=\frac{\phi\times m_{i}(t)}{1+\zeta\times m_{i}(t)}$ (6)

This relationship saturates at *ϕ /ζ* at high human Mf densities and has an initial slope of *ϕ*. Because of this saturation, the development of the parasite in the vector is one of the density regulation mechanisms in the transmission of the parasite.

In case of a constant *foi* (which may be approximated in adults and in the absence of interventions or strong natural fluctuations), the expected equilibrium number of adult worms equals:

$M_{i}=foi_{i}\times(\overline{Tl}-Ti)$ (7)

$\overline{Tl}$is the mean lifespan of the parasite and *Ti* the duration of the immature stage. The lifespan is assumed to vary between parasites according to a Weibull distribution with mean $\overline{Tl}$ and shape-parameter *α_Tl_*. The duration of the immature stage is considered to be invariable. The parasite lifespan not only determines the equilibrium worm-load, but also the rate at which the worm-load declines in case of interruption of transmission.

The dynamics in the Mf density *m* is given by the following expression:

$m_{i}(t)=s\times m_{i}(t-1)+F_{i}(t)\times r_{i}(t)$ (8)

with *s* being the monthly survival of the microfilariae and *r* the number of microfilariae produced by each female worm per month and per 20 μl peripheral blood taken for diagnosis. *F* quantifies the number of adult female worms. On average *F=M/*2, but because the worm-load *M* is a discrete number, particularly at low worm numbers the sex-ratio may deviate from 1:1. The Mf density is defined as the average number of Mf in a 20μl bloodsmear. The actual (discrete) number of Mf counted in the smear is a random selection from a Negative Binomial distribution with mean = *m_i_* and parameter of dispersion *k_m_*.

The Mf production *r* follows from:

$r_{i}(t)=r_{0}\times[1-Rw_{i}(t)]$ (9)

In this expression *r_0_* is the Mf production in the absence of an immune-response and in the presence of at least one adult male in the human host. In case of single-sex infections, Mf production is assumed to be zero. *Rw* quantifies the immune-responsiveness against the adult worms (see below). In case *Rw*=1, the immune-system will completely block the development and production of microfilariae.

In LYMFASIM we assume two mechanisms for the impact of the immune system on the dynamics of the parasite: (1) anti-L3 immunity caused by prolonged exposure to L3-antigens, resulting in a reduction of the success of inoculated L3-larvae to mature and settle in the human body (see equation [2]), and (2) anti-worm immunity caused by the cumulative presence of adult parasites, modelled as a reduction in the fecundity of female worms (equation [9]). Though the way it is modelled in LYMFASIM is debatable, the assumption of these two types of responses is in accordance with the existing knowledge about the regulation of the parasite [[11](#_ENREF_11), [12](#_ENREF_12)].

The first type of response is deduced from the work of Day *et al.* [[13](#_ENREF_13)] who found antibodies to the L3 surface mainly in subjects of 20 years and older, i.e. subjects with the longest history of L3-inoculation. An age-specific increase in the presence of antibodies was also found by Beuria *et al*. [[14](#_ENREF_14)], who further concluded that antibody levels were highly variable between individuals. As regards the anti-worm immunity, it has been suggested that prolonged presence of adult parasites causes a breakdown in tolerance to the parasites, resulting in clearance of microfilariae and progress of disease [[11](#_ENREF_11)]. Whether and to what extent the adult worms or the microfilariae are the target of this response is not clear. In the model we assume that immune-responses cause a reduced fecundity (Mf output).

The mathematical formulation for both types of immune-responses is analogous to a model proposed by Woolhouse [[15](#_ENREF_15)]. The level and dynamics of anti-L3 immunity in person *i* at time *t* is given by:

$Rl_{i}(t)=1-\exp[-\gamma_{l}\times\rho_{l,i}\times Hl_{i}(t)]$ (10)

$Hl_{i}(t)=\beta_{l}\times Hl_{i}(t-1)+mtp_{i}(t)$ (11)

The implication of the anti-L3 immunity *Rl* is given in equation 2. It varies with the ‘experience of L3 infection’, *Hl*, which increases with the number of inoculated L3-larvae (quantified by the monthly transmission potential *mtp*) and which decreases each month with a factor β*_l_*. This decrease represents the decay in specific memory cells or antibodies. The factor γ*_l_* (‘strength of anti-L3 immunity’) translates the experience of L3-infection into an immune-response. Noticing that the immune-responsiveness may vary between individuals even in the case of similar experience of infection, the factor ρ*_l_* is included. This factor varies according to a gamma probability distribution with mean = 1 and shape-parameter α*_l_*. This definition of anti-L3 immunity is analogous to the ‘LL’ model described by Woolhouse [[15](#_ENREF_15)].

The expressions for the anti-worm (anti-fecundity) response are highly similar to those for the anti-L3 response and is analogous to Woolhouse’s ‘AE’ model:

$Rw_{i}(t)=1-\exp[-\gamma_{w}\times\rho_{w,i}\times Hw_{i}(t)]$ (12)

$Hw_{i}(t)=\beta_{w}\times Hw_{i}(t-1)+M_{i}(t)$ (13)

This type of response is driven by the current and past worm-load *M* (experience of worm-infection). The factors β*_w_* (decay of specific antibodies of memory cells), γ*_w_* (‘strength of anti-worm immunity’), and ρ*_w_* (immunity variation; described by a gamma distribution with mean 1 and shape α*_w_*) are thought to be independent from the corresponding factors for the anti-L3 immunity. The impact of anti-worm immunity is given by equation 9.

The factors β*_l_* and β*_w_* determine the duration of the immune-responses after cessation of boosting (inoculation of L3-larvae, presence of worms). In the remainder, we will consider the following transformation of these parameters:

$THl=[\ln(0.5)/\ln(\beta_{l})]/12$ (14)

$THw=[\ln(0.5)/\ln(\beta_{w})]/12$ (15)

These transformations express the half-life (in years) of the experience of infection (*Hl* or *Hw*) in the absence of boosting. We will call this 'immunological memory'.

A list of all model parameters mentioned in this description is provided in Tables 1 and 2. For the quantification of some parameters we use external sources (observations, experiments, literature) or we simply fixed the value (e.g., parameter *v*; see equation [5]). These parameters are listed in Table 1. However the majority will be estimated by fitting the model to the reference data (Table 2).

## Mass treatment coverage and compliance

To simulate the impact of mass drug administration, the user must specify the exact moments of treatment (year, month), the drug or administration regimen applied with its efficacy, the fraction of people treated per round (coverage), and the compliance pattern.

The model considers three posible effect mechanisms: (1) a fraction of adult worms is killed; (2) a fraction of female adult worms is permanently sterilized (i.e. they stop producing mf); and (3) a fraction of mf is killed. The fraction of parasites affected can be constant or can vary according to a chosen probability distribution function.

The effect of treatment on Mf is described as follows:

$m_{i}(t+\varepsilon)=m_{i}(t)\times(1-dm_{i})$ (16)

with *m_i_* the number of microfilariae (scaled to the 20 μL blood volume normally used for diagnosis) at time *t*, treatment occurring between time *t* and *t*+ε, and *dm_i_* the fraction if Mf killed as a result of the treatment. The latter is stochastic variable (<= 1) which varies between persons and between treatments and which is described by a user-defined probability distribution function.

The effect of treatment on adult worms is described by:

$M_{i}(t+\varepsilon)=M_{i}(t)\times(1-dM_{i})$ (17)

with *M_i_* the number of adult worm per individual and *dM_i_* the fractio of worms killed as a result of the treatment. *dM_i_* is again a stochastic variables (<= 1) which varies between persons and between treatments and which is described by a user-defined probability distribution function.

A permanent or temporary reduction in mf production *r_i_(t)* by female parasites in individual *i*, who was treated at time τ, is simulated as follows:

$r_{i}(t)=r_{í}^{'}(t)\times(1-df_{i})\times\left(\frac{t-\tau}{Tr_{i}}\right)^{\sigma}$, for t- τ < *Tr_i_* (18)

$=r_{í}^{'}(t)\times(1-df_{i})$, otherwise (19)

With $r_{í}^{'}(t)$being the Mf production of female worms had person *i* not been treated (the outcome of equation [9], *df_i_* (<= 1) the irreversible reduction of fecundity caused by the treatment, *Tr_i_* the period during which the female parasite recovers from treatment (assuming that immediately after treatment production is zero), and *σ* (>0) a shape parameter which determines how Mf production increases during the recovery period *Tr*: *σ*=1 implies a linear increase, *σ* <1 implies that recovery of productivity is mainly at the end of the period *Tr* (note that *σ* → 0 is equivalent to *Tr* → 0, while *σ* → ∞ implies the total absence of Mf prodution during *Tr*).Both *Tr_i_* and *df_i_* are stochastic variables described by a probability distribution function.

All stochastic variables related to the effects of treatment (*dm*, *dM*, *df*, *Tr*)are by default assumed to be independent and to be generated for each person at each treatment. (The user can also to chose to attribute treament efficacy as a fixed characteristic to an individual, who in that case always responds in the same way to treatment.)

The compliance pattern describes the tendency of persons to participate in repeated treatment rounds. In case of random compliance, all individuals have the same probability to be treated (equal to the fraction covered). In case of systematic compliance, each person in the population is characterized by an invariable compliance factor (a random number between 0 and 1), which results in a treatment probability of either 1 (for compliance factorfcoverage) or 0 (for compliance factor> coverage). Consequently, if coverage is constant over time, some individuals will always be treated while the remaining persons are never treated. In the case of semi-systematic compliance pattern, the compliance factor indicates a person’s tendency to participate. Random numbers define whether an individual is actually treated or not. The latter pattern is presumably most realistic [[16](#_ENREF_16)].

LYMFASIM also allows the simulation of selective treatment. In that case, treatment is only provided to those persons who were Mf positive in the most recent survey (which may take place in the same month as treatment, see below). Coverage and compliance play no role. Vector control can be modelled as a percentage reduction of the monthly biting rate during a specified period. The number of such periods, their duration and the reduction in monthly biting rate can be chosen.

## Vector control

Vector control is modelled as a reduction of the average monthly biting rates during a given period of time. The number of such periods can be chosen. A period of vector control is specified by the year + month of the beginning of the strategy and the year + month of the end of a strategy.

## Surveys

During the simulation, surveys will take place at user-defined timepoints. During a survey, for all simulated individuals the actual number of male and female worms is recorded, and a diagnostic test is simulated to obtain mf counts. It is assumed that Mf are counted in 20μL bloodsmear, or a multiple *φ* of this (taken at the appropriate time during the day in view of circadian periodical appearance in the blood). The Mf count *Mf_i_* for person *i* is then given by:

$Mf_{i}=Negbin(\varphi\times m_{i},k_{m})$ (20)

i.e. the number is assumed to follow a negative binomial distribution with clumping factor *k_m_.* The user can specify other distributions if deemed more appropriate.

## Running the model

Each simulations starts with a predefined initial population with specified size and age distribution, and a short-term external force-of-infection. It has a long burn-in period (typically >100 years) to allow both the population composition and infection levels to establish at a dynamic equilibrium, determined by input parameters. The individual-based structure allows that heterogeneities within the human and parasite population are taken account. The characteristics of individuals are determined by chance and stochastic processes partly determine an individual’s infection load. As a result, the outcome of a single simulation run represents only one of many possible outcomes. Repeated runs will give slightly different results. The variability between runs reflects natural variation in real world settings, conditional on the appropriateness of the model structure. Simulations always have to be repeated to estimate the mean outcome and gain an insight into variability.

# LYMFASIM input: Probability distributions, functions and parameter values

The table below gives a complete overview of model assumption and parameter values used for the simulations in the following manuscript:

Jambulingam P, Subramanian S, de Vlas SJ, Vinubala C, Stolk WA. Mathematical modelling of lymphatic filariasis elimination programs in India: required duration of mass drug administration and post-treatment level of infection indicators. Parasites and Vectors (2016)

| **Parameter** | **Value** | **Source** |
| --- | --- | --- |
| **Human demography** | | |
| *Cumulative survival ($F\left( a \right)$), by age* | | Fixed in [[6](#_ENREF_6)] |
| 0 | 1.000 |  |
| 5 | 0.904 |  |
| 10 | 0.895 |  |
| 15 | 0.888 |  |
| 20 | 0.879 |  |
| 25 | 0.864 |  |
| 30 | 0.849 |  |
| 40 | 0.812 |  |
| 50 | 0.756 |  |
| 90 | 0.000 |  |
| *Fertility rate per woman ($R\left( a \right)$), by age* | | Fixed in [[6](#_ENREF_6)] |
| 0 | 0.000 |  |
| 5 | 0.000 |  |
| 10 | 0.000 |  |
| 15 | 0.000 |  |
| 20 | 0.075 |  |
| 25 | 0.254 |  |
| 30 | 0.222 |  |
| 40 | 0.096 |  |
| 50 | 0.013 |  |
| 90 | 0.000 |  |

| **Parameter** | **Value** | **Source** |
| --- | --- | --- |
| **Transmission of infection** | | |
| *General transmission parameters* |  |  |
| Average mosquito biting rate (m*br*) for adult men | User defined. Varied between simulations |  |
| Transmission probability (v), fraction of the L3 larvae, resulting from a single blood meal, that is released by a mosquito | $v=0.1$ | Fixed in [[6](#_ENREF_6)] |
| Success ratio (*sr*) | $sr=1.03\times{10}^{-3}$ | Fitted in[[6](#_ENREF_6)] |
| *Individual relative exposure to flies* |  |  |
| Variation in by age (*Ea*) | Exposure at birth, E0 = 0.26.  Thereafter, exposure increses linearly with age to reach a maximum 1 at the age *a_max_* of 19.1, from when onwards it remains stable at this level. | Fitted in[[6](#_ENREF_6)] |
| Variation in the individual exposure index *Ei*, due to personal factors (fixed through life) given age and sex | Gamma distribution with mean fixed at 1.0 and shape parameter equal to 1.13 | Fitted in[[6](#_ENREF_6)] |
| *Anti-L3 immunity (triggered by incoming L3 larvae, modifying the probability that the larvae survives and becomes an adult worm)* | |  |
| Average impact of host immunity ($\alpha_{I})$ | $\alpha_{I}=1.07$. Shape-parameter for the gamma-distribution describing individual variation in the ability to develop an anti-L3 immune-response | Fitted in [[6](#_ENREF_6)] |
| Strength of immunological memory ${(\rho}_{l})$ | $\rho_{I}=5.89\times{10}^{-5}$. Scale parameter for sigmoid function relating strength of anti-L3 immunity to experience of infection by L3 | Fitted in [[6](#_ENREF_6)] |
| Duration of immunological memory ${(TH}_{l})$ | *TH_l_=9.6* years. Period in which strength of anti-L3 immune response is halved in the absence of boosting by L3 | Fitted in [[6](#_ENREF_6)] |
| *Anti-fecundity immunity was not used* | |  |
| **Life history and productivity of the parasite in the human host** | | |
| Average worm lifespan (*Tl*) | 10.2 years | Fitted in[[6](#_ENREF_6)] |
| Variation in worm lifespan (*α_Tl_*) | Weibull distribution with shape 2.0.  A value of *α_Tl_* = 1 means an exponential distribution, which is often implicitly assumed in mathematical models. *α_Tl_* > 1 implies less variation. | Fixed, based on the estimated variation in adult worm lifepan for *Onchocerca volvulus* [[17](#_ENREF_17), [18](#_ENREF_18)] |
| Duration of immature stage of the parasite in human host | 8 months | Fixed [[19](#_ENREF_19)] |
| No. of Mf produced per female parasite per month  per 20 ml peripheral blood in the absence of immunereactions  and in the presence of at least 1 male worm (*r_0_*) | 0.606 per 20 *µl* blood. Assumed to be independent of parasite age | Fitted in[[6](#_ENREF_6)] |
| Monthly survival of the microfilariae, fraction (*s*) | 0.9 | Fixed, based on[[20](#_ENREF_20)] |
| Polygamy | Assumed to be polygamous. All female worms produce mf in the presence of at least one male worm. | Assumption (in the absence of data, it is widely assumed that intestinal and filarial nematodes are polygamous[[21](#_ENREF_21)]) |
|  |  |  |
| **Uptake of infection by the vector** | | |
| Number of L3 larvae developing in mosquitoes, as a function of the mf density in the human blood | Hyperbolic saturating function with parameters *ϕ* = 0.09, and *ζ* = 0.013 (equation 6) | [[10](#_ENREF_10)] |
| **Mass treatment coverage** | | |
| Coverage ($C_{w}$) | User defined, e.g. $C_{w}=65\%.$ Assumed to be constant over different rounds of MDA |  |
| Pattern of compliance | Individual’s compliance with MDA is assumed to be Partially-systematic, as described in [[16](#_ENREF_16)]. i.e. it is neither completely random (where each person has the same chance to get treated in each round) nor completely systematic (where all individuals either take all or none of the treatments), but somewhere in between. Variation between age and sex groups in compliance was not considered |  |
| **Timing and characteristics of treatment** |  |  |
| No. of MDA rounds | User defined |  |
| Timing of treatments | User defined |  |

| **Parameter** | **Value** | **Source** |
| --- | --- | --- |
| **Drug treatment efficacy** | | |
| Single dose DEC | *dm*  (fraction of mf killed by treatment): 70%  *dM* (fraction of adult worms killed):50%  *df_i_* (the irreversible reduction of fecundity caused by the treatment): 0%  *Tr_i_* (the period during which the female parasite recovers from treatment): NA  Variation: All treatment effects are assumed to be constant in this study, so that there is no variation between individuals and treatments | Assumption, see [[22](#_ENREF_22)] for justification |
| Single dose DEC+ALB | *dm* (fraction of mf killed by treatment): 70%  *dM* (fraction of adult worms killed):65%  *df_i_* (the irreversible reduction of fecundity caused by the treatment): 0%  *Tr_i_* (the period during which the female parasite recovers from treatment): NA  Variation: All treatment effects are assumed to be constant in this study, so that there is no variation between individuals and treatments | Assumption, see [[22](#_ENREF_22), [23](#_ENREF_23)] for justification |
| **Vector control** | | |
| Timing | Not used. |  |
| Effectiveness | Not used. |  |
| **Epidemiological surveillance** |  |  |
| Volume of blood used for Mf-count | 20 microliter | Assumption |
| Variability in measured host load of infective material (Mf per 20 *µl* blood) | *k_m_*=0.345. Overdispersion parameter of the Negative Binomial distribution describing the variation in Mf counts in blood smears for an individual | Fitted in[[6](#_ENREF_6)] |
| No. and timing of surveys | User defined. The number of surveys, and specific moment of surveys, and the timing of first survey if subsequent surveys are conducted at regular intervals can be specified. |  |

# References

1. Plaisier AP, van Oortmarssen GJ, Habbema JD, Remme J, Alley ES. ONCHOSIM: a model and computer simulation program for the transmission and control of onchocerciasis. Comput Methods Programs Biomed. 1990;31(1):43-56.

2. De Vlas SJ, Van Oortmarssen GJ, Gryseels B, Polderman AM, Plaisier AP, Habbema JD. SCHISTOSIM: a microsimulation model for the epidemiology and control of schistosomiasis. Am J Trop Med Hyg. 1996;55(5 Suppl):170-5.

3. Coffeng LE, Bakker R, Montresor A, de Vlas SJ. Feasibility of controlling hookworm infection through preventive chemotherapy: a simulation study using the individual-based WORMSIM modelling framework. Parasit Vectors. 2015;8:541.

4. Habbema JDF, De Vlas SJ, Plaisier AP, Van Oortmarssen GJ. The microsimulation approach to epidemiologic modeling of helminthic infections, with special reference to schistosomiasis. Am J Trop Med Hyg. 1996;55(5 Suppl):165-9.

5. Plaisier AP, Subramanian S, Das PK, Souza W, Lapa T, Furtado AF et al. The LYMFASIM simulation program for modeling lymphatic filariasis and its control. Methods of Information in Medicine. 1998;37:97-108.

6. Subramanian S, Stolk WA, Ramaiah KD, Plaisier AP, Krishnamoorthy K, Van Oortmarssen GJ et al. The dynamics of *Wuchereria bancrofti* infection: a model-based analysis of longitudinal data from Pondicherry, India. Parasitology. 2004;128(Pt 5):467-82.

7. Stolk WA, Stone C, de Vlas SJ. Modelling lymphatic filariasis transmission and control: modelling frameworks, lessons learned and future directions. Adv Parasitol. 2015;87:249-91.

8. Brabin L. Sex differentials in susceptibility to lymphatic filariasis and implications for maternal child immunity. Epidemiol Infect. 1990;105(2):335-53.

9. Rajagopalan PK, Das PK, Subramanian S, Vanamail P, Ramaiah KD. Bancroftian filariasis in Pondicherry, south India: 1. Pre-control epidemiological observations. Epidemiol Infect. 1989;103(3):685-92.

10. Subramanian S, Krishnamoorthy K, Ramaiah KD, Habbema JDF, Das PK, Plaisier AP. The relationship between microfilarial load in the human host and uptake and development of *Wuchereria bancrofti* microfilariae by *Culex quinquefasciatus*: a study under natural conditions. Parasitology. 1998;116:243-55.

11. Maizels RM, Lawrence RA. Immunological tolerance: the key feature in human filariasis? Parasitology Today. 1991;7(10):271-6.

12. Ottesen EA. The Wellcome Trust Lecture. Infection and disease in lymphatic filariasis: an immunological perspective. Parasitology. 1992;104(Suppl):S71-9.

13. Day KP, Grenfell B, Spark R, Kazura JW, Alpers MP. Age specific patterns of change in the dynamics of *Wuchereria bancrofti* infection in Papua New Guinea. Am J Trop Med Hyg. 1991;44(5):518-27.

14. Beuria MK, Bal M, Dash AP, Das MK. Age-related prevalence of antibodies to infective larvae of *Wuchereria bancrofti* in normal individuals from a filaria-endemic region. J Biosci. 1995;20(2):167-74.

15. Woolhouse ME. A theoretical framework for the immunoepidemiology of helminth infection. Parasite Immunol. 1992;14(6):563-78.

16. Plaisier AP, Stolk WA, van Oortmarssen GJ, Habbema JD. Effectiveness of annual ivermectin treatment for *Wuchereria bancrofti* infection. Parasitol Today. 2000;16(7):298-302.

17. Plaisier AP, van Oortmarssen GJ, Remme J, Habbema JD. The reproductive lifespan of *Onchocerca volvulus* in West African savanna. Acta Trop. 1991;48(4):271-84.

18. Remme J, De Sole G, van Oortmarssen GJ. The predicted and observed decline in onchocerciasis infection during 14 years of successful control of Simulium spp. in west Africa. Bull World Health Organ. 1990;68(3):331-9.

19. WHO. Lymphatic filariasis: the disease and its control. Fifth report of the WHO Expert Committee on Filariasis. World Health Organ Tech Rep Ser. 1992;821:1-71.

20. Plaisier AP, Cao WC, van Oortmarssen GJ, Habbema JD. Efficacy of ivermectin in the treatment of *Wuchereria bancrofti* infection: a model-based analysis of trial results. Parasitology. 1999;119(Pt 4):385-94.

21. Anderson RM, May RM. Helminth infections of humans: mathematical models, population dynamics, and control. Adv Parasitol. 1985;24:1-101.

22. Stolk WA, De Vlas SJ, Habbema JDF. Anti-*Wolbachia* treatment for lymphatic filariasis. Lancet. 2005;365(9477):2067-8.

23. Stolk WA, ten Bosch QA, de Vlas SJ, Fischer PU, Weil GJ, Goldman AS. Modeling the impact and costs of semiannual mass drug administration for accelerated elimination of lymphatic filariasis. PLoS Negl Trop Dis. 2013;7(1):e1984.
