# Supplementary material for: Mathematical modelling of lymphatic filariasis elimination programmes in India: required duration of mass drug administration and post-treatment level of infection indicators
Source: Parasit Vectors. 2016 Sep 13;9(1):501. doi: 10.1186/s13071-016-1768-y (PMC5022201; doi:10.1186/s13071-016-1768-y)

Supplementary figure S1: Model-predicted Mf and Ag prevalence in the population aged 5 years and above and 6-7 year old children, measured 1 year after the last round of MDA with the total duration of MDA specified as in Table 3. Results are presented for 12 settings, varying with respect to the assumed monthly biting rate and coverage achieved in MDA. Antigenaemia is assumed to be detectable if an individual carries at least one female worm (hypothesis 2). See legend to figure 4 for additional information regarding the interpretation of the boxplots.

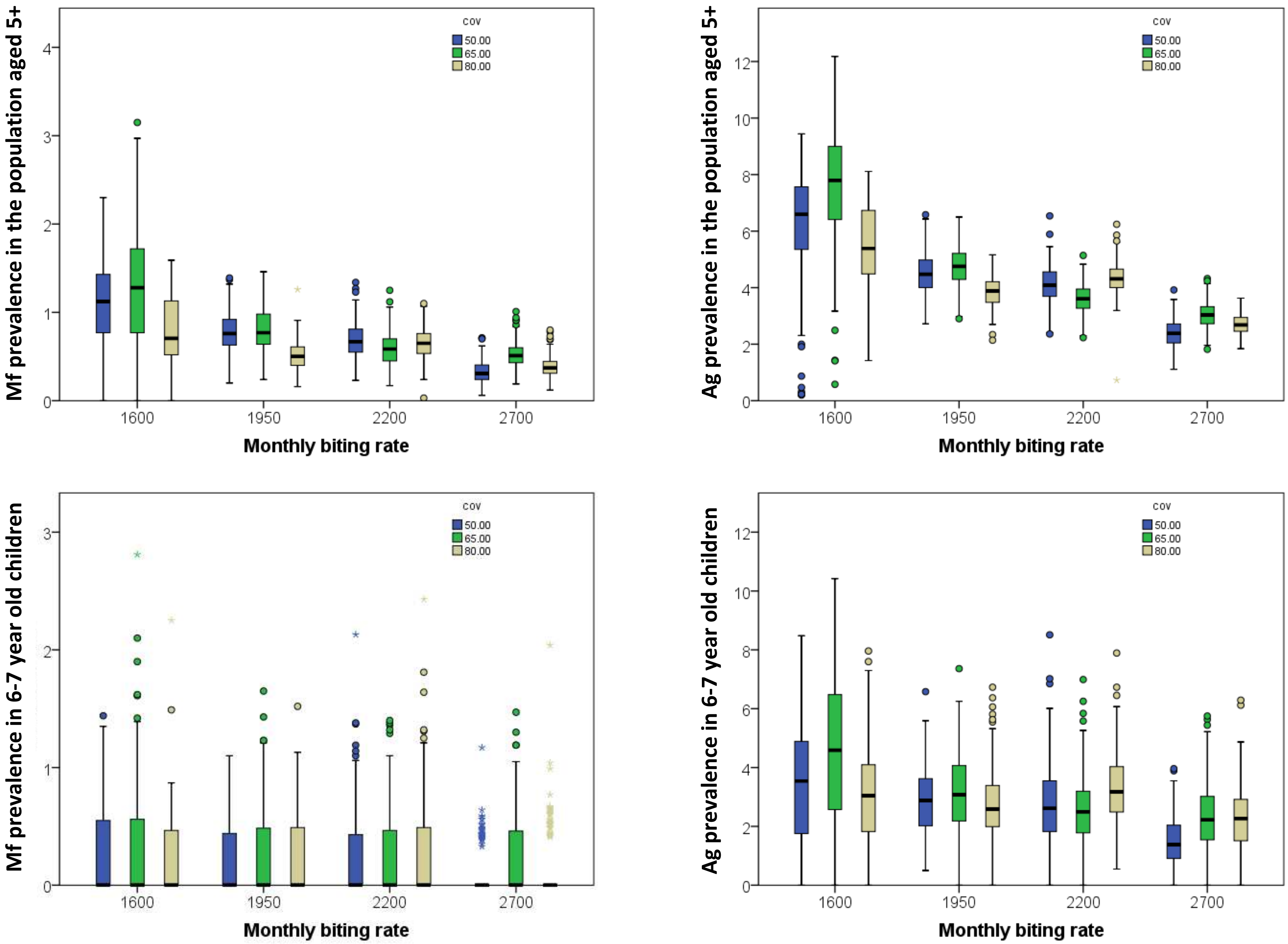

Supplement: Additional file 5: Figure S1. — For clustered boxplots by mbr and coverage. (PDF 112 kb) [file 13071_2016_1768_MOESM5_ESM.pdf]
